# Peruvian origin and global invasions of five continents by the highly damaging agricultural pest *Liriomyza huidobrensis* (Diptera: Agromyzidae)

**DOI:** 10.1111/eva.13702

**Published:** 2024-10-21

**Authors:** Sonja J. Scheffer, Matthew L. Lewis, Norma Mujica, Charles MacVean, Helga Blanco‐Metzler, Ravindra C. Joshi, Frode Jacobsen

**Affiliations:** ^1^ Systematic Entomology Laboratory USDA‐ARS Beltsville Maryland USA; ^2^ Universidad Nacional Agraria La Molina La Molina Lima Peru; ^3^ Kinsley School of Engineering, Sciences and Technology York College of Pennsylvania York Pennsylvania USA; ^4^ Crop Protection Research Centre University of Costa Rica San Jose Costa Rica; ^5^ Philippine Rice Research Institute, Maligaya Science City of Muñoz Nueva Ecija Philippines

**Keywords:** bioinvasions, bottleneck, geographic origin, leaf mining fly, leafminer, phylogeography

## Abstract

Identification of the geographic origin of invasive species can be critical to effective management and amelioration of negative impacts in the introduced range. *Liriomyza huidobrensis* is a polyphagous leafmining fly that is a devastating pest of many vegetable and floriculture crops around the world. Considered native to South and possibly Central America, *L. huidobrensis* became invasive in the 1980s and has since spread to at least 30 countries on five continents. We used phylogeographic analysis of over 2 kb of mitochondrial cytochrome oxidase I and II sequence data from 403 field‐collected specimens from both native and introduced populations to investigate the geographic origins of invasive *L. huidobrensis* worldwide. Within South America, there was substantial genetic variation, as well as the strong phylogeographic structure typical of a native range. In contrast, leafminers from the introduced range and Central America all contained little genetic variation and shared the same small set of haplotypes. These haplotypes trace to Peru as the ultimate geographic origin of invasive populations. Central America is rejected as part of the original geographic range of *L. huidobrensis.* Within Peru, the primary export region of Lima shared an extremely similar pattern of reduced haplotype variation to the invasive populations. An additional 18 specimens collected at US ports of entry did not share the same haplotype profile as contemporary invasive populations, raising perplexing questions on global pathways and establishment success in this species.

## 1 INTRODUCTION

With the ever‐increasing global movement of people and goods, the number of invasive species and the damage they cause has risen dramatically (Hulme, 2009; Meyerson & Mooney, 2007; Westphal et al., 2008). Invasive species are currently considered one of the main threats to biodiversity, ecosystem function, and food production (Cook et al., 2011; Lee, 2002; Paini et al., 2016; Pimentel et al., 2005, 2007; Pyšek & Richardson, 2010). Estimates of damage due to invasive arthropods are as high as $70 billion per year (Bradshaw et al., 2016). In general, about half of invasive arthropod species are phytophagous, many of which are highly damaging agricultural pests (Liebhold et al., 2016).

Determining the native range and place of origin of invasive pest populations is critical for managing invasive species (Estoup & Guillemaud, 2010; Guillemaud et al., 2011; Le Roux & Wieczorek, 2009; Navia et al., 2005). With this information, scientists and resource managers gain access to important biological and ecological information such as host specificity, life cycle, and insecticide efficacy/resistance. Biological control programs typically rely on searching for natural enemies within native source populations, which serve as important reservoirs of natural enemies of potential biocontrol use (Center et al., 2012). In addition, understanding the origin and pathways of invasion provides a framework for developing new control and quarantine procedures to restrict further spread (Estoup & Guillemaud, 2010; Liebhold et al., 2016). Finally, determining the origin population(s) provides a framework for investigation of the ecology, population biology, and evolutionary dynamics of invasions (Dlugosch & Parker, 2008; Handley et al., 2011).

Molecular markers and population genetics have proven valuable in determining the original range of introduced populations (Handley et al., 2011; Le Roux & Wieczorek, 2009; Roderick, 2004; Scheffer & Grissell, 2003). Introductions typically involve a genetic bottleneck, where an introduced population may contain only a subset of the genetic variation present in the native range (Ahern et al., 2009; Dlugosch & Parker, 2008; Edelaar et al., 2015; Scheffer et al., 2006; Scheffer & Lewis, 2005, 2006). When genetic variation in native populations is phylogeographically structured, it can be possible to determine the specific source location/region of the introduced populations (Avise, 1991; Le Roux & Wieczorek, 2009; Scheffer & Grissell, 2003). However, recolonizations of the same locations, as well as populations resulting from multiple founding populations, can lead to patterns that may obscure the genetic patterns of the initial invasions. Therefore, for assessment of early patterns and processes involved with the movement of invasive species, samples collected contemporaneously with the invasion(s) are the most useful.

*Liriomyza huidobrensis* Blanchard (Diptera: Agromyzidae) is a highly polyphagous leafmining fly that feeds on more than 365 plant species in 49 plant families (Spencer, 1973; Weintraub et al., 2017). More than 78% of its recorded host plants are agricultural crops. Eggs are laid within host leaves via oviposition punctures made by females, and the resulting larvae ingest mesophyll as they feed within the leaf, leaving a conspicuous tunnel or “mine” as they feed. Large populations of these leafminers cause multiple types of damage to infested plants, which may lead to plant death and complete crop loss (Parrella, 1987; Shepard & Braun, 1998; Weintraub et al., 2017). *Liriomyza huidobrensis*, along with several of its congeners, were historically considered “secondary” pests—those that, although present, do not cause substantial economic damage (Reitz et al., 2013; Spencer, 1973). However, heavy insecticide use can eliminate the parasitoid wasps that control leafminer numbers and drive the evolution of insecticide resistance (Chavez & Raman, 1987; Reitz et al., 2013). In the 1970s and 1980s, outbreak populations of *L. huidobrensis* in Peru developed due to intensive spraying of insecticides against the co‐occurring potato moth, *Tuta absoluta* (Meyrick) (Chavez & Raman, 1987; Weintraub et al., 2017). At nearly the same time as outbreak populations arose in Peru, populations of *L. huidobrensis* became problematic in Central America, where *L. huidobrensis* had been previously unknown. In addition, several countries in South America were important in the early global fresh vegetable and flower trade, which was associated with early introductions to Europe (Rodríguez‐Castañeda et al., 2017; Weintraub et al., 2017).

Since the late 1980s, *L. huidobrensis* has spread from its native range in South America and possibly Central America to at least 30 countries on five continents (Weintraub et al., 2017; Weintraub & Horowitz, 1995). The global movement of *L. huidobrensis* primarily occurred via the international shipment of infested vegetable and flower crops (Mujica et al., 2017; Reitz et al., 2013; Weintraub et al., 2017). Although this movement occurred coincident with the buildup of the insecticide‐resistant outbreak populations within Peru, other locations such as Central America or other South American countries may have been the origin, or one of the origins, of invasive populations. Central America is often cited as part of the native range of *L. huidobrensis*, but whether Central American populations represent previously undetected native populations or whether they have resulted from introductions from South America is not clear (Weintraub et al., 2017).

The primary goal of this project was to use mitochondrial cytochrome oxidase I (COI) and cytochrome oxidase II (COII) DNA sequence data to investigate genetic variation and phylogeographic structure within both native and introduced *L. huidobrensis* populations. Our samples were collected shortly after various occurrences of introductions in the 1990s. Specifically, we ask (1) whether sequence data from the mitochondrial COI and COII region can differentiate introduced from native populations, (2) whether variation within introduced populations traced to a single or multiple regions of the native populations, and (3) whether genetic patterns of global spread are consistent with historical information on invasions by *L. huidobrensis*.

## 2 MATERIALS AND METHODS

Specimens were collected by rearing, sweep‐netting, or removal from sticky traps and included adults, larvae, and pupae depending on the collecting method. Samples were collected from 29 crops and other plant species. Additional samples were obtained by trapping or other means from presumptive hosts, all of which have been previously reported to be hosts of *L. huidobrensis* (Weintraub et al., 2017).

Samples were obtained from 16 countries and 26 locations across seven major geographic regions: Asia (China, Indonesia, Malaysia, Philippines, Sri Lanka); Central America (Costa Rica, Guatemala, Panama); Europe (Italy); the Middle East (Israel); North America (Canada); South America (Argentina, Brazil, Colombia, Ecuador, Peru); and Africa (South Africa) (Table 1). The number of populations sampled within countries ranged from one to four. The amount of time between the first report of introduced *L. huidobrensis* populations within a country and the collection of our specimens averaged 3.6 years. With the exception of samples from Peru and Ecuador, populations within single countries are combined for analyses and discussion.

**TABLE 1:** Country, ample size, year of invasion, collection date, collection locations, and host affiliations for specimens in this study.

Country	*N*	Invasion year	Sample year	Sample locations	Host plants
*South America*	173				
Argentina	9	Native	2000	Cordoba	*Beta vulgaris* [beet]
Colombia	22	Native	2000	Ile Del Cauca, Pradera	*Phaseolus vulgaris* [kidney bean]
Ecuador	49	Native	1999, 2002, 2003, 2003	Carchi, Chimborazo, Pinchincha	*Solanum tuberosum* [potato] *Gypsophila* sp. [flower]
Peru	93	Native	1998, 1999, 2001	Canete, Huanacayo, Huspedero, Lima	*Allium cepa* [shallot], *Apium graveolens* [celery], *Lactuca sativa* [lettuce], *Lupinus mutabilis* [Andean lupine], *Medicago sativa* [alfalfa], *Phaseolus vulgaris* [green beans], *Pisum sativum* [pea], *Raphanus raphanistrum* [radish], *Solanum lycopersicum* [tomato], *Solanum tuberosum* [potato], *Spinacia oleracea* [spinach], *Apium graveolens* [celery], *Vicia faba* [fava bean]
*Central America*	66				
Costa Rica	17	1980s	2005	Coronado, Poas Alajuela	*Brassica oleracea* [broccoli], *Eruca vesicaria* [arugula]
Guatemala	42	1980s	1998, 2000	Chimaltenango	*Pisum sativum* [snowpea], *Vicia faba* [fava bean], “weed”
Panama	7	1980s	2004	Cero Punta	*Brassica rapa* [mustard]
*Introduced*	164				
Canada	9	1998	2000	Ontario, Simcoeco, and York Counties	Swept
China	9	1993	2001	Bejing colony	Colony
Indonesia	17	1994	1999	West Java	*Solanum tuberosum* [potato]
Israel	13	1992	1999, 2000		*Apium graveolens* [celery], *Lactuca sativa* [lettuce], *Solanum tuberosum* [potato]
Italy	9	1991	2000	Bolonga colony	Colony
Malaysia	2	(1994?)	1998	Pahang	*Capsicum annuum* [peppers], *Solanum lycopersicum* [tomato]
Philippines	60	1999	2000, 2001	Balili, Banaue Prov., Benguet Prov., Mountain Prov.	*Allium cepa*, *Apium graveolens* [celery], *Arctium lappa* [greater burdock], *Brassica juncea* [mustard], *Brassica oleracea* [cabbage or broccoli], *Brassica rapa* [bok choy], *Chrysanthemum*, *Cucurbita pepo* [winter squash and pumpkin], *Daucus carota* [carrot], *Lactuca sativa* [lettuce], *Phaseolis vulgaris* [string beans], *Pisum sativum* [pea], *Sida* sp., *Solanum lycopersicum* [tomato], *Sonchus oleraceus*[sowthistle], *Vigna unguiculata* [pole beans]
South Africa	31	1999	2000	Sanvelt Region, Vivo Region	*Solanum tuberosum* [potato]
Sri Lanka	14	(1993?)	1998	Galpalama, Eliya	*Allium ampeloprasum* [leeks], *Beta vulgaris* [beet], *Brassica oleracea* [cabbage], *Brassica rapa* [mustard], *Chrysanthemum* sp. [mums], *emilia*
*Interceptions*	18				
Colombia	8	Native	2000, 2001, 2008	Not recorded	*Artemisia*, “aster”, *Chrysanthemum*
Ecuador	2	Native	2001	Not recorded	*Eustoma*, *Gypsophila*
Kenya	2	(1990s?)	2008	Not recorded	*Ranunculus*
Netherlands	6	1980s	2008	Not recorded	*Ranunculus*

*Note*: Intercepted samples were those intercepted by USDA‐APHIS‐PPQ agents at US Ports of Entry.

We also obtained *L. huidobrensis* specimens that had been intercepted at US Ports of Entry by USDA Plant Protection and Quarantine agents during the years 2000–2008 (Table 1). These samples were collected during formal inspections of commercial vegetable and flower material being imported into the United States. With the import documentation, there is a source country listed, although whether the specimens came from established field populations in those countries cannot be confirmed. For this reason, these specimens are not included in the global analyses of the distribution and geographic variation in haplotypes. However, they are included in the haplotype network and phylogeographic analyses, see below. The source countries of record for these specimens were Colombia, Ecuador, Kenya, and The Netherlands.

Upon collection, specimens were placed into various concentrations of ethanol depending on the collector, but once in the primary laboratory, they were switched to 95% and stored at −80°C. Data were collected for 2–93 individuals from each country, with a mean of 25. Our primary global dataset consisted of 403 individuals (Table S1). An additional 18 individuals were collected from US border interceptions (Table S1).

Total nucleic acids were extracted from individual specimens using the DNeasy DNA Extraction kit (QIAGEN Inc., Valencia, CA). The mitochondrial cytochrome oxidase I and II and the intervening tRNA‐leucine region (2400 bp) were amplified with primers TY‐J‐1461 and TD‐N‐3862 (Table S2) using a BioRad Tetrad 2 (Bio‐Rad, Hercules, CA, USA) with a touchdown amplification program: initial denaturation at 92°C for 2 min, followed by touchdown cycles from 58° to 46°C (10 s at 92°C, 10 s at 58–46°C, 2 min at 72°C), 29 cycles of 10 s at 92°C, 10 s at 45°C, 2 min at 72°C, and a final extension step for 10 min at 72°C. The PCR amplicon spanned almost the entire mitochondrial cytochrome oxidase I and II genes as well as the intervening leucine t‐RNA gene (Table S2).

The PCR product was enzymatically purified using ExoSAP‐IT (Affymetrix, Santa Clara, CA, USA). Sequencing reactions were carried out using Big Dye Terminator v3.1 Sequencing kits (Applied Biosystems, Foster City, CA) using both internal and external primers (Table 3). Sequencing reactions were cleaned with ethanol precipitation and analyzed on an ABI 3730XL automated DNA sequencer.

For each individual, a contig spanning the COI/tRNA/COII region was assembled using the software package Sequencher (Gene Codes Corp., Ann Arbor, MI). The final collection of contigs was aligned using Genieous Prime 2021.2.2 (https://www.geneious.com). Each contig was inspected visually for any ambiguities or sites lacking reverse complement corroboration. Individuals with any ambiguous or unconfirmed sites were removed from the project. To avoid missing data due to uneven contig lengths, the final alignment was trimmed at both ends to a length of 2181 bp.

This resulted in a final alignment of 403 consensus sequences from specimens collected within countries and a second alignment of 18 sequences from port interceptions. All sequences have been submitted to GenBank under accession numbers OR971906–OR972281 (Table S1).

### 2.1 Analyses

Population statistics for each country were obtained using R (Team RC, 2019), RStudio (Team R, 2019), and the packages ape (Paradis & Schliep, 2019) and pegas (Paradis, 2010). We calculated haplotype diversity, nucleotide diversity, and maximum pairwise divergence for each country. We used PopART to conduct analyses of molecular variation (AMOVAs) to explore population structure across three populations in Peru and three in Ecuador. Because of the almost complete lack of haplotype sharing among South American countries (see Section 3), an AMOVA involving the entire South American dataset is not appropriate.

For haplotype network and phylogeographic analyses, the sequence alignment was reduced to contain only a single representative of each haplotype. Haplotypes from the specimens intercepted at the US border were included in this dataset. We constructed a minimum‐spanning haplotype network using PopART (Leigh & Bryant, 2015). A neighbor‐joining (NJ) analysis with 1000 bootstrap replications was carried out using Geneious (http://www.geneious.com). Maximum likelihood (ML) analysis was carried out in the web version IQ‐TREE (Trifinopoulos et al., 2016) using automated model choice and default settings. In both cases, the outgroup taxon was *L. langei* Frick, the closely related sister species of *L. huidobrensis* (Scheffer, 2000; Scheffer & Lewis, 2001). Pairwise mitochondrial distances to *L. langei* ranged from 5.4% to 5.8%.

## 3 RESULTS

### 3.1 Haplotype diversity and distributions

**FIGURE 1:**
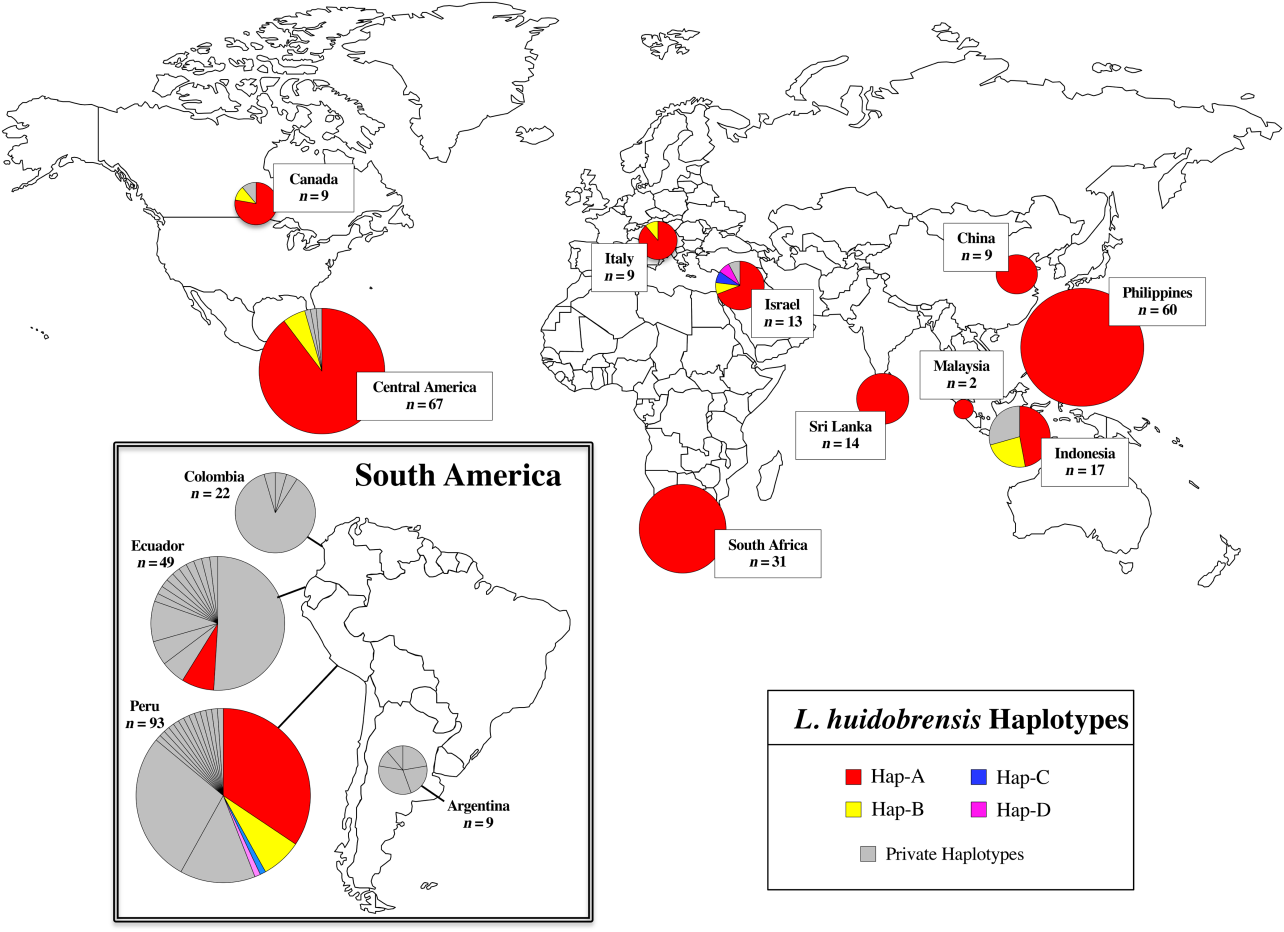
Global distribution of collection sites and haplotype composition of populations of *Liriomyza huidobrensis*. Each circle represents pooled samples for each country. Sample sizes are shown within boxes; in addition, circles are approximately scaled for population size. Slices within each circle represent haplotypes present in that particular location and indicate relative abundance. All private haplotypes (those found only in a single country) are shown in grey. Haplotypes shared between two or more countries are indicated in color.

From the main sample of 403 specimens, we found 47 mitochondrial haplotypes with variation at 72 (3.3%) of the 2181 bp sites. Of these haplotypes, 43 (91%) were private and found only within a single country (Table 2, Figure 1). Populations from South America accounted for 37 (86%) of these private haplotypes (Table 2). Pairwise distances across *L. huidobrensis* sequences ranged from 0 to 0.73%.

**TABLE 2:** Distribution of private and invasive (“shared”) haplotypes in various locations, maximum pairwise distances, haplotype, and nucleotide diversities.

Country	*N*	# Total haps	# Private haps	MAX PWD %	Hap diversity	Nuc. diversity	# Inv. Hap‐A	# Inv. Hap‐B	# Inv. Hap‐C	# Inv. Hap‐D	% Invasive haps
*South America*											
Argentina	9	5	5	0.183	0.8611	0.000917					0
Colombia	22	8	4	0.504	0.4586	0.000862					0
Ecuador	49	14	13	0.779	0.7074	0.003149	4				7.8
Peru	93	19	15	0.275	0.7892	0.001018	32	7	1	1	43.6
SA total	173										
*Central America*											
Costa Rica	17	3	2	0.092	0.2279	0.000107	15				88.2
Guatemala	42	3	1	0.183	0.2195	0.000264	37	4			97.6
Panama	7	1	0	0	0	0	7				100
CA total	66										
*Introduced*											
Canada	9	3	1	0.183	0.4167	0.000407	7	1			88.9
China	9	1	0	0	0	0	9				100
Indonesia	17	3	1	0.183	0.6765	0.000728	8	4			70.6
Israel	13	5	1	0.275	0.5385	0.000882	9	1	1	1	92.3
Italy	9	2	0	0.137	0.2222	0.000305	8	1			100
Malaysia	2	1	0	0	0	0	2				100
Philippines	60	1	0	0	0	0	60				100
South Africa	31	1	0	0	0	0	31				100
Sri Lanka	14	1	0	0	0	0	14				100
Intr. total	164										
Total *N*	403						243	18	2	2	

Four of the 47 haplotypes found in the study were shared between two or more countries. These haplotypes (“Haps A–D”) were also present in at least one introduced population. We refer to these as “invasive” haplotypes (Table 2, Figure 1). An additional six unique haplotypes were found in introduced and Central American populations, carried by 4% of the overall introduced population (Table 2, Figure 1). These haplotypes are not shared between countries, do not provide information on geographic origins, and are referred to as “private” rather than “invasive” in the text below.

Hap‐A was the most abundant and geographically widespread invasive haplotype, found in 243 (60%) of the 403 individuals collected within countries overall (Table 2, Figure 1). It dominated eight of the nine introduced populations and all three Central American populations with 207 of the 232 (89%) individuals collected outside of South America carrying this haplotype. This haplotype was associated with all 27 crops in the introduced range (Table 3). Within South America, Hap‐A was carried by 32 of the 93 individuals (34%) from Peru and four of 49 individuals (8%) from Ecuador. The three remaining invasive haplotypes, Haps B–D, were much less abundant globally, with none carried by more than 18 individuals in the study. Within South America, these three haplotypes were only present in Peru (Table 2, Figure 1). All the hosts of Haps B–D also served as hosts of Hap‐A (Table 3).

**TABLE 3:** Invasive haplotypes from this study, their associated host plants, and locations where they occur given our sampling.

Haplotype	*N*	Host plants (locations)
Hap‐A	234	Alfalfa (Peru), arugula (Costa Rica), fava beans (Guatemala), beets (Sri Lanka), broccoli (Costa Rica), burdock (Philippines), cabbage (Philippines, Sri Lanka), carrots?? (Philippines), celery (Israel, Peru, Philippines), chrysanthemums (Philippines, Sri Lanka), gagatang (Philippines), onion (Philippines, Sri Lanka), green beans (Peru, Philippines), lettuce (Israel, Peru, Philippines), lupine (Peru), mustard (Panama, Philippines, Sri Lanka), peas (Peru, Philippines), peppers (Malasia), pechay (Philippines), potato (Ecuador, Indonesia, Israel, Peru), shallots (Philippines), snowpeas (Guatemala), spinach (Peru), tomato (Malaysia, Peru, Philippines), zucchini (Philippines); also greenhouses and colonies (Canada, China, Italy) – gobo (Philippines)
Hap‐B	18	Fava beans (Guatemala, Peru), lupine (Peru), potato (Indonesia, Israel), snowpea (Guatemala), colony, greenhouse (Canada, Italy)
Hap‐C	2	Fava bean (Peru), celery (Israel)
Hap‐D	2	Potato (Israel, Peru)

*Note*: See Table 1 for Latin names of host plants.

Most of the populations known to be introduced consisted almost entirely of a single or subset of invasive haplotypes, dominated by Hap‐A (Figure 1). Some countries possessed a single private haplotype not found in other locations, but only in Indonesia was a private haplotype carried by more than 15% of the population. The genetic makeup of populations within the three Central American countries was very similar to that of the introduced populations, with 63 of 66 (95%) Central American specimens carrying the invasive haplotypes Hap‐A and Hap‐B. The remaining three individuals each carried a singleton haplotype which was all only a single mutational step from Hap‐A (see below).

**FIGURE 2:**
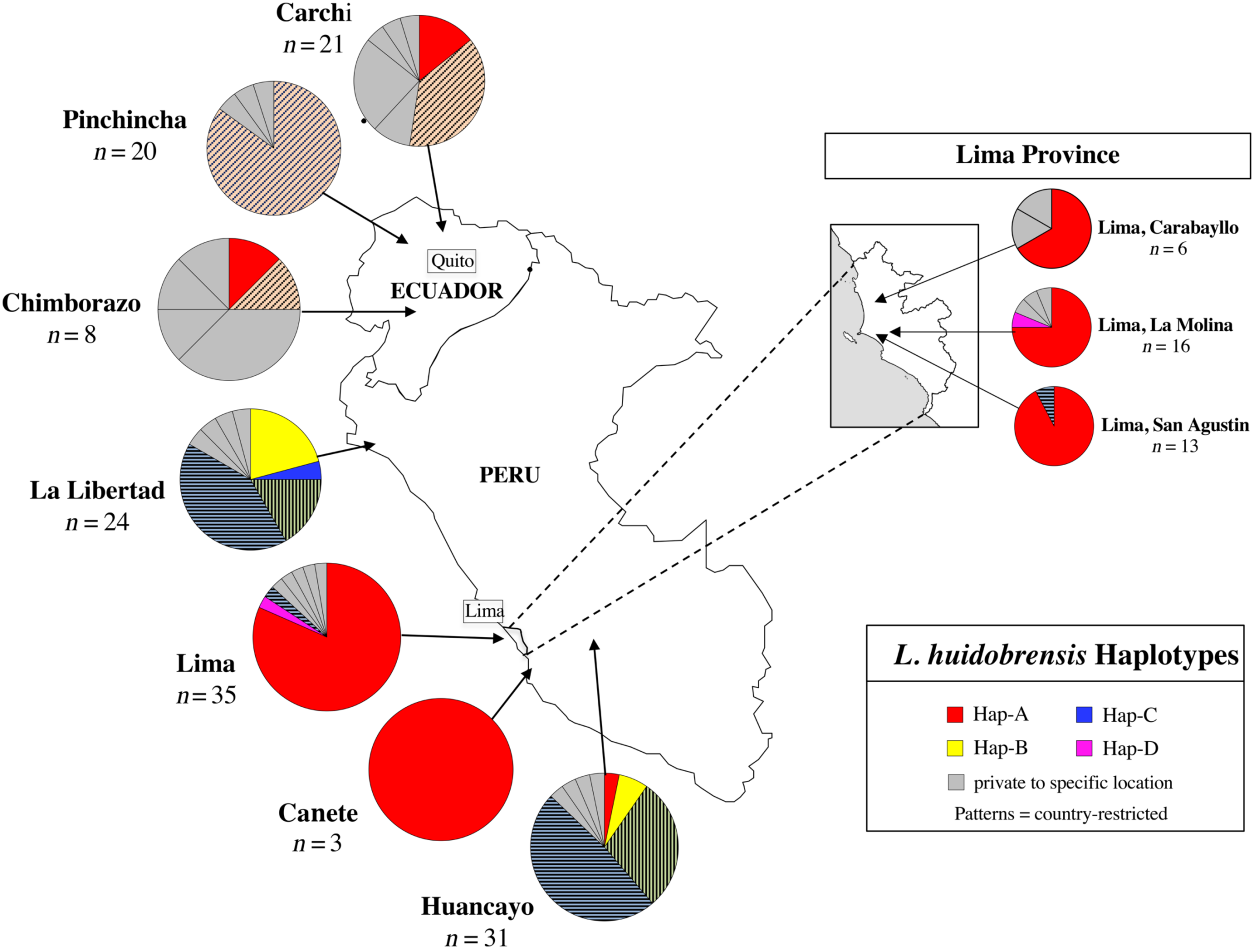
Regional distribution of private and invasive haplotypes in Ecuador and Peru. Details as in Figure 1. Non‐invasive haplotypes shared among locations are shown in a variety of patterns and colors.

Within South America, the only sharing of haplotypes between countries was the occurrence of Hap‐A in Peru and Ecuador. Otherwise, populations within each country were comprised of unique haplotypes. All four invasive haplotypes were present in Peru, and these made up 41 of the 93 (44%) sampled individuals (Table 2). Within Peru, there was significant within‐country variation in haplotype structure across the three sampled populations (Table 4, Figure 2). The population from the lowland Lima Region was made up of 88% of invasive haplotypes, primarily Hap‐A. This population also contained individuals with both Hap‐C and Hap‐D, as well as five specimens each with a unique haplotype. The overall pattern of haplotype composition and frequencies of this regional Lima population is highly similar to those of the globally invasive populations.

**TABLE 4:** Molecular analyses of variance (MANOVA) for the three primary populations sampled in Peru: Lima, La Libertad, and Huancayo.

Source	df	Sum of squares	Sigma 2	%Variation	*p* Value
Among populations	2	157.181	1.653	48.63758	<0.001
Within populations	87	157.066	1.745	51.36242	
Total	89	314.227			

*Note*: FI = 0.48638, *p* < 0.001.

In contrast to the Lima population, Hap‐A was not present in La Libertad and was uncommon in Huancayo (Figure 2). In the La Libertad region of Peru, two invasive haplotypes were present, Hap‐B and Hap‐C, while Hap‐A and Hap‐B were present in the highland Huancayo region. In addition, the La Libertad and Huancayo populations shared two Peruvian‐restricted haplotypes, and each contained four private alleles (Figure 2). Results from the AMOVA found significant differences in haplotype structure across the three populations (Table 4).

Similarly, in Ecuador, the three sampled populations differed in haplotype structure (Table 5, Figure 2). Only four individuals carrying Hap‐A were found within Ecuador, and these were at low frequency in Carchi and Chimborazo. No other invasive haplotypes were present in the country. Only one haplotype was present in all three populations, while the remaining 12 haplotypes were unique to single populations (Figure 2).

**TABLE 5:** Molecular analyses of variance (MANOVA) for the three populations sampled in Ecuador: Carchi, Pinchincha, and Chimborazo.

Source	df	Sum of squares	Sigma 2	%Variation	*p* Value
Among populations	2	725.694	13.579	31.04479	<0.001
Within populations	46	1387.408	30.161	68.95521	
Total	48	2113.102			

*Note*: FI = 0.31045, *p* < 0.001.

From the 18 specimens intercepted at US Ports of Entry from six presumptive countries, we found 10 haplotypes. Five of these haplotypes were unique and not present in other individuals in the study. Hap‐A was the only invasive haplotype found in the intercepted flies and was only found in specimens coming from Kenya and The Netherlands, both of which are introduced locations. None of the samples intercepted from the native range of Colombia or Ecuador carried any of the four invasive haplotypes.

**FIGURE 3:**
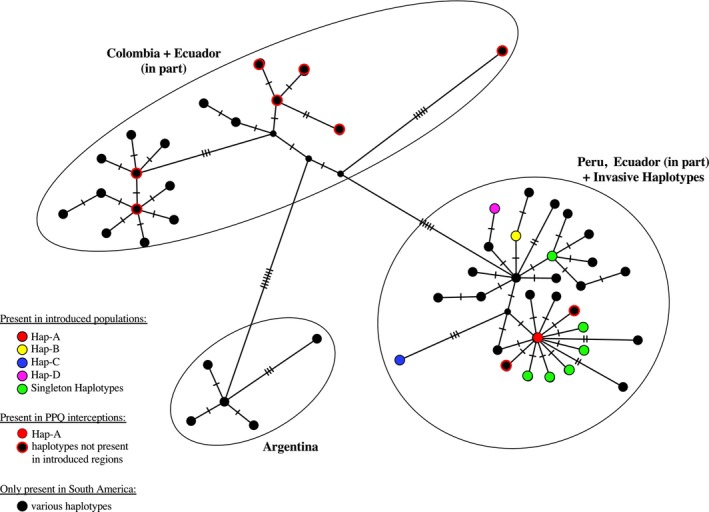
Haplotype network for all haplotypes including invasive haplotypes, singletons from introduced populations, and those intercepted at US Ports of Entry. Invasive haplotypes Haps A–D found in the introduced location are shown in color as in Figures 1 and 2. Green circles are singleton haplotypes found in introduced populations. Black circles edged with red indicate haplotypes from individuals intercepted at US ports of entry.

The minimum‐spanning haplotype network of all haplotypes found three distinct clusters (Figure 3). One consisted of all specimens from Argentina. A second comprised all Colombian and some Ecuadorian samples. The third cluster was made up of all Peruvians, the remaining Ecuadorians, and all haplotypes found in introduced locations. This included the four invasive haplotypes as well as all six introduced country‐restricted haplotypes. The three main clusters were separated by long mutational branches. Five of the six country‐restricted haplotypes found in introduced locations were only a single mutational step from Hap‐A, including all three singleton haplotypes from Central America. From the intercepted specimens, only three of the 10 haplotypes belonged to the Peru/Ecuador cluster. These were Hap‐A and two haplotypes only one mutational step from it (Figure 3). The remaining seven haplotypes in intercepted specimens were highly distinct from the Peruvian and related haplotypes (Figure 3).

### 3.2 Phylogeographic structure

**FIGURE 4:**
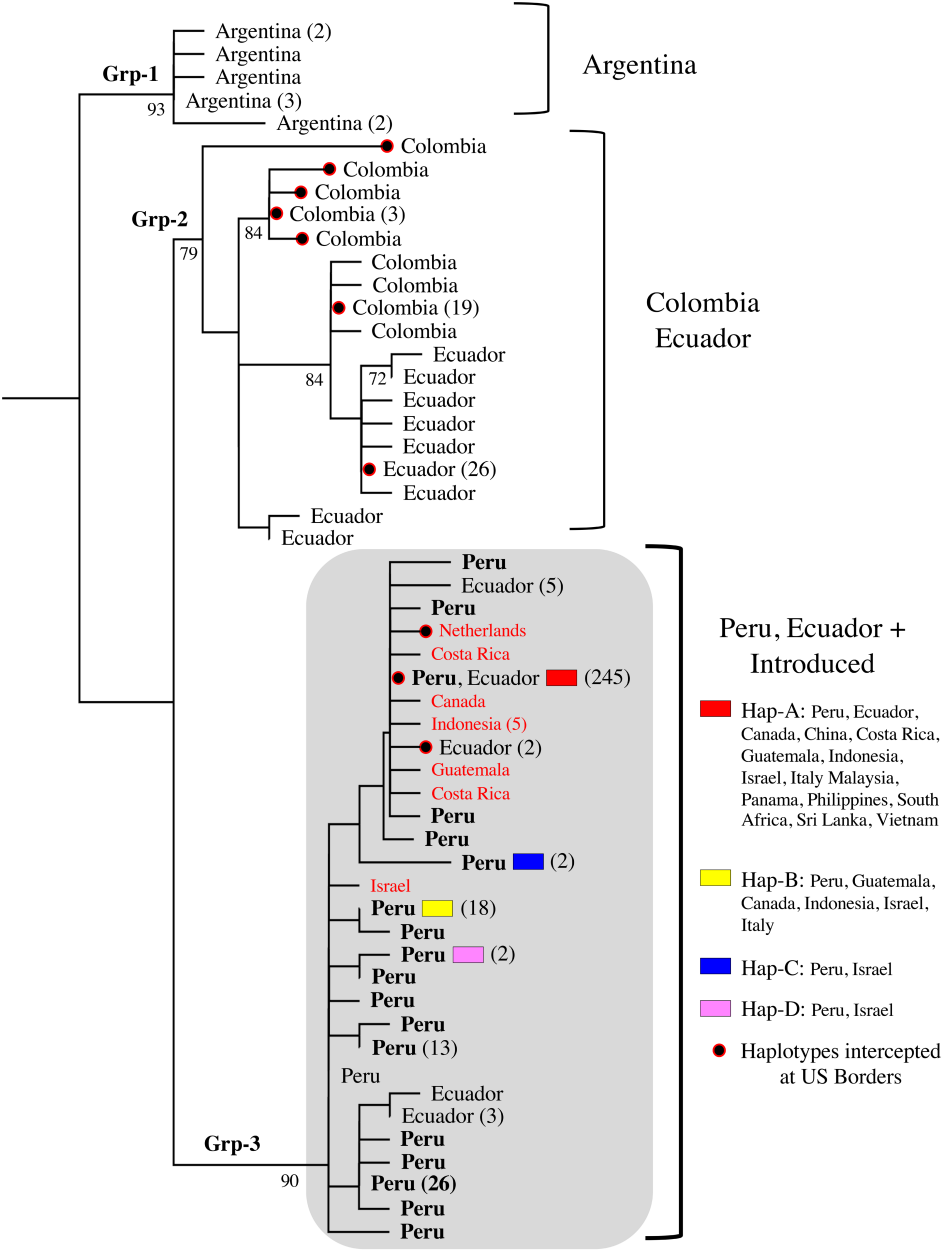
Maximum likelihood analysis of global *Liriomyza huidobrensis* haplotypes. Invasive haplotypes Haps A–D found in the introduced location are shown in color as in previous figures. The number of individuals carrying each haplotype is shown in parentheses when more than one. Country names in red type indicate introduced populations. Black circles edged with red indicate haplotypes from individuals intercepted at US ports of entry. Bootstrap support values for Groups 1, 2, and 3 are presented at each node.

In both NJ and ML analyses, we included all haplotypes from the study, including the intercepted specimens, for a dataset of 53 haplotypes from 421 specimens. The topologies were the same with the exception of a few within population placements involving single mutations. The ML tree is presented here, with bootstrap values above 70 shown below‐supported branches (Figure 4). When considering only samples from the native range, there was a strong clustering of haplotypes by geography (Figure 4). Group 1 consisted of the five haplotypes from Argentina flies that formed a well‐supported, monophyletic group that was sister to the rest of the samples. The remaining haplotypes came out in two sister groups. Group 2 consisted of all nine of the Colombian haplotypes as well as nine of the 13 Ecuadorian haplotypes. Neither country within this clade formed a monophyletic group. Seven of the 10 haplotypes intercepted at US Ports of Entry belonged to this group (Figure 4).

Group 3 was the largest group and comprised 30 haplotypes from 323 individuals (Figure 4). This group included all Peruvian haplotypes, all the invasive haplotypes (Haps A–D), and all additional haplotypes found outside of South America. This included the haplotypes from the 66 flies from Central America. Three of the 10 haplotypes from US Ports of Entry, including Hap‐A, belonged to this group. Several Ecuadorian samples also belonged to this group. There was no evidence of geographic structure within Group 3 (Figure 4).

The 18 specimens collected from US Ports of Entry did not form a unified cluster; instead, they were dispersed within Group 2 and Group 3 (Figure 4). All intercepted individuals carrying Hap‐A were from the introduced locations in Kenya and the Netherlands in 2008. Individuals from the native range, Ecuador, and Colombia, carried haplotypes that clustered with other specimens from their country of record.

## 4 DISCUSSION

A number of factors influence the ability of an agricultural pest to successfully invade new locations (Liebhold et al., 2016; Lockwood et al., 2005; Simberloff, 2009). These include abundance, small size, pesticide resistance, broad host range, and cryptic locations on transported agricultural products. These contribute to heavy propagule pressure, the continuing onslaught of potential invaders such that some eventually are successfully introduced (Liebhold et al., 2016; Simberloff, 2009). *Liriomyza huidobrensis* possesses all of these characteristics: outbreak populations of small, cryptic, endophytic larvae located inside a broad range of produce and ornamental crop species that are widely shipped around the world. The inadvertent, anthropogenic spread of this species from the Americas to dozens of countries on five continents occurred in fewer than 30 years (Weintraub et al., 2017).

### 4.1 Haplotype structure in native and introduced ranges

Our analyses of global *L. huidobrensis* populations confirm South America as the native range of the invasive *L. huidobrensis* populations. Haplotype distributions, frequencies, and relationships all support South America as the original source. South American populations were substantially more diverse than introduced populations and showed strong phylogeographic structure consistent with the geographic proximities of the South American countries. Other than four individuals carrying Hap‐A in Ecuador, none of the remaining 40 South American haplotypes were shared among countries in the native range. The only other *L. huidobrensis* sequence data available from South America is from Brazil (Sousa et al., 2022). As would be expected from the present results, the Brazilian sequence data differed from all of the of the 53 haplotypes found in the present study (Scheffer, pers. obs.). Within South America, the phylogeographic pattern is one of highly structured, long‐term populations having limited gene flow across substantial geographic distances (Avise, 2009; Wright, 1943).

In contrast to the high levels of haplotype diversity and structure present within *L. huidobrensis* across its native range, genetic patterns in the introduced populations were nearly uniform across the globe. Only a fraction of the number of haplotypes were present than in the native range. Almost all introduced populations were dominated by Hap‐A, which was carried by approximately 90% of individuals in these populations. Previous studies have also shown a lack of mitochondrial variation in introduced *L. huidobrensis* populations (He et al., 2002; Xu, Coquilleau, et al., 2021). He et al. (2002) sequenced 53 *L. huidobrensis* specimens from nine crops throughout Yunnan Province, China, and found only a single haplotype. Xu, Coquilleau, et al. (2021) sequenced 45 specimens from Australia, Indonesia, and Kenya and also found only a single haplotype. The haplotypes from both studies are identical to Hap‐A across their length, but both studies used a much shorter piece of the COI/COII region (~700 bp) which precludes full comparison. Without the full length of the 2181 bp region, we cannot be certain whether any nucleotide base changes might be present. In any case, the pattern of reduced genetic variation and a lack of phylogeographic structure in introduced populations is typical of invasive organisms and is generally interpreted as an indication of a history of founder events and population bottlenecks during colonization (Nei et al., 1975; Slade & Moritz, 1998).

### 4.2 Peruvian origin of invasive haplotypes

Our results clearly identify Peru as the ultimate origin of all invasive *L. huidobrensis* populations sampled in this and other studies (He et al., 2002; Xu, Coquilleau, et al., 2021). Within South America, all invasive haplotypes were found in Peruvian populations and clustered within Group 3. Furthermore, all additional haplotypes present within invasive populations belonged to Group 3 along with all haplotypes collected within Peru. With the exception of the four individuals carrying Hap‐A in Ecuador, there is no molecular evidence supporting the involvement of any additional primary source region(s) in the ultimate origin of our sampled invasive populations.

Within Peru, there were considerable differences among populations in genetic structure and haplotype composition. We found an equivalent pattern of regional differences in population structure in Ecuador. Within Peru, we found that samples from the Lima Region had a genetic signature that was extremely similar to that of the introduced populations, with 82% of the individuals carrying Hap‐A. This is in contrast to the make‐up of the two other Peruvian populations that were both dominated by a mixture of haplotypes and bore little resemblance to invasive populations in either haplotype composition or frequencies (Figure 2).

Our identification of Peru as the ultimate source region for the invasive populations is consistent with historical records that the initial global spread of *L. huidobrensis* occurred contemporaneously with the emergence of outbreak populations of insecticide‐resistant *L. huidobrensis* in Peru (Chavez & Raman, 1987; Reitz & Trumble, 2002; Weintraub et al., 2017). During the initial global spread of *L. huidobrensis*, Lima was the primary point of export of flowers and produce originating in Peru. In fact, the Lima population(s) are so similar in mitochondrial composition to global populations, with these data we are unable to assess whether populations leaving Lima underwent severe bottlenecks. Although the Lima Region appears very likely to be the initial source population for the export of invasive *L. huidobrensis*, we do not yet have exhaustive sampling within the country. Most crucial is the question of whether other yet unsampled populations in Peru also share the genetic signature of invasive populations, such that invasive haplotypes in the Lima Region may be themselves the result of or influenced by introductions involving infested plant material from other Peruvian locations.

Although our data on border interceptions are not extensive, we analyzed eight specimens from South America that were collected during the same time period during which most of the field samples were collected. These samples are from Colombia and Ecuador and carried seven different haplotypes, none of which were present in introduced populations. These sequences clustered with other Colombian and Ecuadorian samples in Group 2 and did not cluster with any sequences from introduced populations in Group 3. This dramatic difference between the haplotype composition of US border interceptions and that of established global populations occurring at the same time is perplexing. It may be that pathways of potential spread, involving native populations from Colombia and Ecuador were globally limited and primarily involved exports to the United States or other countries having stringent quarantine procedures in place at the time. Alternatively, the observed discrepancy between border interceptions and global patterns could reflect differential colonization success by flies carrying Peruvian invasive haplotypes. This could be possibly due to greater propagule pressure leaving Peru, or descendent populations, resulting in the high number of colonizers in combination with their well‐developed insecticide resistance. In fact, the likely insecticide‐resistant flies leaving both Peru and any subsequent introduced locations may have been preadapted for invasion success, as has been suggested for other invasive species (Le Roux & Wieczorek, 2009).

Although Peru is the ultimate origin of invasive populations, it is almost certainly not the case that all introductions came directly from Peru. Published records indicate that while there were several introductions from Peru to one or more locations, these new populations in turn may have served as a bridgehead source of subsequent introductions (Weintraub et al., 2017). From quarantine interceptions and published reports, likely bridgehead locations include Central America (particularly Costa Rica) and The Netherlands, both heavily involved in the flower trade at the time (Weintraub et al., 2017). In addition, the current distribution of *L. huidobrensis* is not entirely due to discrete introductions from distant populations. Regional spread due to local commodity transport as well as the movement of the flies themselves, must account for much of the geographic footprint we see today.

### 4.3 Status of populations in Central America

Central America has, at times, been considered part of the native range of *L. huidobrensis* (Weintraub et al., 2017). Our data clearly show that this is not the case and that populations in Central America are the result of one or more introductions. First, the genetic patterns found in Central America are essentially the same as those in introduced populations in the rest of the world (Figure 1). Central American populations had similarly reduced variation and were composed primarily of invasive haplotypes, with only a few private haplotypes. These private haplotypes are only a single mutation from Hap‐A and cluster in Group 3, along with all 18 of the Peruvian haplotypes and four of 13 from Ecuador. Given the strong phylogeographic structure found across South America, it is extremely unlikely that Central America would share invasive and related haplotypes with Peru/Ecuador without human intervention.

Consistent with the interpretation that populations in Central America are the result of one or more introductions is that in Spencer's (1973) extensive review of economically important agromyzid pests, no Central American countries were listed as part of the range of *L. huidobrensis*, although this species has been known from South America since at least the 1920s (Blanchard, 1926). It was not until 1983 that *L. huidobrensis* was reported from Central America, coinciding with the initial global spread of invasive populations (Spencer, 1983). In addition, populations in Guatemala are notorious for feeding almost exclusively on agricultural crops rather than native hosts, an unusual pattern for a native insect (MacVean & Perez, 1996; Weintraub et al., 2017).

### 4.4 Ongoing introductions

*Liriomyza huidobrensis* has continued to invade new regions and countries. Most notably, established populations have been found recently in India and Australia (Mhatre et al., 2022; Mulholland et al., 2022; Weintraub et al., 2017; Xu, Coquilleau, et al., 2021).

Interestingly, *L. huidobrensis* has not become established within the United States despite a long history of quarantine interceptions at US borders (Scheffer et al., 2014; Weintraub et al., 2017; this study). Because of substantial agricultural production of vegetable and flower crops, California and Florida have been considered the most likely locations within the United States to be vulnerable to an introduction by *L. huidobrensis*. However, temperature modeling for southern Florida has suggested that this is not true for this typically hot region (Rodríguez‐Castañeda et al., 2017). The sole report of “*L. huidobrensis*” established in Florida was a small and short‐lived population in 1981 attributed to the importation of nursery stock from California (Poe & Montz, 1981, reported in Parrella & Keil, 1984, Steck, 1996). This record undoubtedly refers to *L. langei* Frick, which is a very similar pest species common in California not recognized as distinct from *L. huidobrensis* at that time (Scheffer, 2000; Scheffer & Lewis, 2001).

Determination of whether invasive *L. huidobrensis* is present in California has been complicated by the widespread presence there of *L. langei*. *Liriomyza langei* is also a damaging polyphagous leafminer and has mines and external morphological features that are essentially indistinguishable from those of *L. huidobrensis* (Spencer, 1973; but see Lonsdale, 2011). In 2000, Morgan et al. (2000) found RFLP frequency differences between central and southern populations of *L. langei* (as “*L. huidobrensis*”) in California, suggesting the possibility that the more damaging central populations represented a recently established invasive *L. huidobrensis* population from outside the United States. However, analysis of mitochondrial sequence data from colonies representing these central and southern populations found them to be nearly identical, and sequences from both populations clustered tightly within a well‐supported and highly distinct clade, now recognized as *L. langei* (Scheffer, 2000; Scheffer & Lewis, 2001). A recent molecular survey and analysis of 664 field‐caught adult leafminers matching the *langei*/*huidobrensis* morphological description in California found that all represented *L. langei* and that *L. huidobrensis* was not present (Scheffer et al., 2014). To date, *L. langei* has not been found to be established outside of the United States. In the absence of data to the contrary, it can be reasonably assumed that established populations within California in both the past and present represent *L. langei* while those in the rest of the world are *L. huidobrensis*. However, molecular data, preferably sequence data, should be used to obtain unambiguous identifications of specimens involved in experimental, systematic, management, or quarantine situations.

In the years since the early introductions, it is likely that the global pathways of *L. huidobrensis* colonizations have evolved alongside changing patterns of trade in vegetable and horticultural commodities, driven by increased globalization and the emergence of new markets. Continuing global movement of *L. huidobrensis* since the original invasions has implications for management strategies and the further evolution of invasive populations. Most importantly, new genetic input due to recolonizations of existing invasive populations by novel and distinct source populations may introduce new genetic variation involving adaptive traits important to successful pest biology, such as insecticide resistance, broadened host use, or increased climate tolerances. In addition to the importation of novel adaptive genes and genetic structure, the input of new variation via mixing of discrete populations could result in a general increase in overall genetic variability available for new adaptive responses to ongoing and future selection.

### 4.5 Invasion dynamics

While mitochondrial data can provide extraordinary information on the geographic and ecological distribution of genetic variation (e.g., the field of phylogeography; Avise, 1991), it typically lacks the number of informative sites necessary for studies of invasion dynamics. In addition, it is uniparentally inherited and haploid thus precluding the most informative statistical methods for population genetics. Although we have been able to show that the ultimate origin of global *L. huidobrensis* is Peru, the few and extensively shared mitochondrial haplotypes in the introduced populations do not allow us to determine the pathways of global spread, including whether individual invasive populations are the result of a single introduction or multiple introductions involving mitochondrially similar populations. Other important questions involving invasion dynamics that we cannot explore with our current dataset include those relating to bottlenecks, population sizes, expansions, and natural selection. However, these can be suitably addressed with genomic data. In the case of *L. huidobrensis*, genomic DNAs of the samples in the present study are still available for analysis using a population genomics approach to investigate processes occurring in the initial stages of global invasions. Comparison of such data with equivalents from *L. huidobrensis* collected in the present will provide an exciting window into the invasion dynamics that have occurred during the first 30 years of global movement by this destructive species.

## 5 CONCLUSION

The present study provides strong evidence of the ultimate origin of global samples of invasive populations of *L. huidobrensis* in Peru, consistent with the history of large outbreaks and the evolution of insecticide resistance there. These results are based on extensive sampling and an unusually large piece of mitochondrial DNA allowing the detection of more informative haplotypes than would be possible with a DNA‐barcoding approach. Most samples in this study were collected during 1998–2001 from established populations shortly after introductions. Thus, the results presented here offer a valuable indication of the initial patterns of *L. huidobrensis* invasions. In combination with extensive global samples, our data provide a substantial framework for future research investigating the ecology and evolutionary biology of global invasions in *L. huidobrensis*.

Direct comparison and analyses of new genetic data with those presented here are expected to provide important further information on dynamic patterns of genetic variation in *L. huidobrensis*. It is certain that genome‐wide molecular approaches will be useful in more precisely determining both the history of past introductions in *L. huidobrensis* and the evolutionary genetics/genomics of current invasive populations (see North et al., 2021). Of considerable interest will be the determination of the degree of recolonization and genetic mixing of populations and the effect this has on adaptation to diverse habitats and conditions.

## CONFLICT OF INTEREST STATEMENT

The authors declare no conflicts of interest.

## Supporting information


Table S1.
Table S2.

## Data Availability

All sequences have been deposited in GenBank under accession numbers OR971906–OR972281. The haplotype alignment and associated information are available at: doi:10.5061/dryad.tqjq2bw6z.
