# Supplementary material for: Peruvian origin and global invasions of five continents by the highly damaging agricultural pest Liriomyza huidobrensis (Diptera: Agromyzidae)
Source: Evol Appl. 2024 Oct 21;17(10):e13702. doi: 10.1111/eva.13702 (PMC11493104; doi:10.1111/eva.13702)
Supplement: Supplementary file 1 — Table S1. Table S2. [file EVA-17-e13702-s001.docx]

Suppl. Table 1. Specimen data including DNA extraction code, country of origin, location, plant associate (reared or trapped), haplotype name, and GenBank accession number. Excluding invasive haplotypes, mitochondrial haplotype codes are defined as follows. The first set of capital letters refer to where the haplotype was found: ARG=Argentina, CAN=Canada, COL=Columbia, CR=Costa Rica, ECU=Ecuador, GUA=Guatemala, IND=Indonesia, ISR=Israel, NETH=Netherlands, and PE=Peru. “PPQ” indicates US border interception. “Shared” indicates that more than one specimen in the country carried that haplotype with N indicating the number of individuals. “Single’ means that the sequence is unique across the entire study.

| **Extract Code** | **Country** | **Location** | **Plant Associate** | **Haplotype Name** | **Genbank #** |
| --- | --- | --- | --- | --- | --- |
| AG2318 | Peru | Huancayo | *Vicia faba* | PE Shared-2 N26 | OR971906 |
| AG2319 | Peru | Huancayo | *Vicia faba* | PE AG2319 Single | OR971907 |
| AG2320 | Peru | Huancayo | *Vicia faba* | PE Shared-1 N13 | OR971908 |
| AG2321 | Peru | Huancayo | *Vicia faba* | PE Shared-2 N26 | OR971909 |
| AG2322 | Peru | Huancayo | *Vicia faba* | PE Shared-2 N26 | OR971910 |
| AG2323 | Peru | Huancayo | *Vicia faba* | PE Shared-2 N26 | OR971911 |
| AG2324 | Peru | Huancayo | *Vicia faba* | Inv Hap-B N18 | OR971912 |
| AG2325 | Peru | Huancayo | *Vicia faba* | PE AG2325 Single | OR971913 |
| AG2326 | Peru | Huancayo | *Vicia faba* | Inv Hap-B N18 | OR971914 |
| AG2327 | Peru | Huancayo | *Vicia faba* | PE Shared-1 N13 | OR971915 |
| AG2328 | Peru | Huancayo | *Vicia faba* | PE Shared-1 N13 | OR971916 |
| AG2329 | Peru | Huancayo | *Vicia faba* | PE Shared-2 N26 | OR971917 |
| AG2342 | Peru | Huancayo | *Vicia faba* | PE Shared-2 N26 | OR971918 |
| AG2343 | Peru | Huancayo | *Vicia faba* | PE Shared-2 N26 | OR971919 |
| AG2344 | Peru | Huancayo | *Vicia faba* | PE Shared-2 N26 | OR971920 |
| AG2345 | Peru | Huancayo | *Vicia faba* | PE Shared-2 N26 | OR971921 |
| AG2346 | Peru | Huancayo | *Vicia faba* | PE Shared-1 N13 | OR971922 |
| AG2347 | Peru | Huancayo | *Vicia faba* | PE Shared-1 N13 | OR971923 |
| AG2349 | Peru | Huancayo | *Vicia faba* | PE Shared-1 N13 | OR971924 |
| AG2351 | Peru | Huancayo | *Vicia faba* | PE Shared-2 N26 | OR971925 |
| AG2352 | Peru | Huancayo | *Vicia faba* | PE AG2352 Single | OR971926 |
| AG2353 | Peru | Huancayo | *Vicia faba* | PE Shared-1 N13 | OR971927 |
| AG2367 | Peru | La Libertad | *Lupinus mutabilis* | PE AG2367 Single | OR971928 |
| AG2369 | Peru | La Libertad | *Lupinus mutabilis* | PE Shared-2 N26 | OR971929 |
| AG2370 | Peru | La Libertad | *Lupinus mutabilis* | PE AG2370 Single | OR971930 |
| AG2371 | Peru | La Libertad | *Lupinus mutabilis* | PE Shared-1 N13 | OR971931 |
| AG2372 | Peru | La Libertad | *Lupinus mutabilis* | PE AG2372 Single | OR971932 |
| AG2373 | Peru | La Libertad | *Lupinus mutabilis* | PE Shared-2 N26 | OR971933 |
| AG2374 | Peru | La Libertad | *Lupinus mutabilis* | PE Shared-2 N26 | OR971934 |
| AG2376 | Peru | La Libertad | *Lupinus mutabilis* | Inv Hap-B N18 | OR971935 |
| AG2377 | Peru | La Libertad | *Lupinus mutabilis* | Inv Hap-B N18 | OR971936 |
| AG2378 | Peru | La Libertad | *Lupinus mutabilis* | PE Shared-2 N26 | OR971937 |
| AG2379 | Peru | La Libertad | *Lupinus mutabilis* | Inv Hap-B N18 | OR971938 |
| AG2380 | Peru | La Libertad | *Lupinus mutabilis* | PE Shared-2 N26 | OR971939 |
| AG2382 | Peru | La Libertad | *Lupinus mutabilis* | Inv Hap-B N18 | OR971940 |
| AG2383 | Peru | Huancayo | *Lupinus mutabilis* | PE Shared-2 N26 | OR971941 |
| AG2384 | Peru | Huancayo | *Lupinus mutabilis* | PE Shared-2 N26 | OR971942 |
| AG2385 | Peru | Huancayo | *Lupinus mutabilis* | PE Shared-1 N13 | OR971943 |
| AG2386 | Peru | Huancayo | *Lupinus mutabilis* | PE AG2386 Single | OR971944 |
| AG2387 | Peru | Huancayo | *Lupinus mutabilis* | PE Shared-1 N13 | OR971945 |
| AG2389 | Peru | Huancayo | *Lupinus mutabilis* | PE Shared-2 N26 | OR971946 |
| LC1 | Colombia | USDA-APHIS PPQ | *Chrysanthemum* sp. | COL Shared-1 N19 | OR971947 |
| LC2 | Colombia | USDA-APHIS PPQ | "aster" | COL LC-2 Single | OR971948 |
| LC3 | Colombia | USDA-APHIS PPQ | *Chrysanthemum* sp. | PPQ Shared-1 N3 | OR971949 |
| LC4 | Colombia | USDA-APHIS PPQ | "aster" | COL LC-4 Single | OR971950 |
| LHD101 | Guatemala | Chimaltenango | *Vicia faba* | Inv Hap-A N245 | OR971951 |
| LHD102 | Guatemala | Chimaltenango | *Vicia faba* | Inv Hap-A N245 | OR971952 |
| LHD103 | Guatemala | Chimaltenango | *Vicia faba* | Inv Hap-A N245 | OR971953 |
| LHD104 | Guatemala | Chimaltenango | *Vicia faba* | Inv Hap-A N245 | OR971954 |
| LHD105 | Guatemala | Chimaltenango | *Vicia faba* | Inv Hap-A N245 | OR971955 |
| LHD106 | Guatemala | Chimaltenango | *Vicia faba* | Inv Hap-A N245 | OR971956 |
| LHD107 | Guatemala | Chimaltenango | *Vicia faba* | Inv Hap-A N245 | OR971957 |
| LHD108 | Guatemala | Chimaltenango | *Vicia faba* | Inv Hap-A N245 | OR971958 |
| LHD109 | Guatemala | Chimaltenango | *Vicia faba* | Inv Hap-A N245 | OR971959 |
| LHD110 | Guatemala | Chimaltenango | *Vicia faba* | Inv Hap-A N245 | OR971960 |
| LHD112 | Guatemala | Chimaltenango | *Vicia faba* | Inv Hap-A N245 | OR971961 |
| LHD113 | Guatemala | Chimaltenango | *Pisum sativum* var. *saccharatum* | Inv Hap-A N245 | OR971962 |
| LHD114 | Guatemala | Chimaltenango | *Pisum sativum* var. *saccharatum* | Inv Hap-A N245 | OR971963 |
| LHD115 | Guatemala | Chimaltenango | *Pisum sativum* var. *saccharatum* | Inv Hap-A N245 | OR971964 |
| LHD116 | Guatemala | Chimaltenango | *Pisum sativum* var. *saccharatum* | Inv Hap-A N245 | OR971965 |
| LHD117 | Guatemala | Chimaltenango | *Pisum sativum* var. *saccharatum* | Inv Hap-A N245 | OR971966 |
| LHD118 | Guatemala | Chimaltenango | *Pisum sativum* var. *saccharatum* | Inv Hap-A N245 | OR971967 |
| LHD119 | Guatemala | Chimaltenango | *Pisum sativum* var. *saccharatum* | Inv Hap-A N245 | OR971968 |
| LHD120 | Guatemala | Chimaltenango | *Pisum sativum* var. *saccharatum* | Inv Hap-B N18 | OR971969 |
| LHD121 | Guatemala | Chimaltenango | *Pisum sativum* var. *saccharatum* | Inv Hap-A N245 | OR971970 |
| LHD122 | Guatemala | Chimaltenango | *Pisum sativum* var. *saccharatum* | Inv Hap-A N245 | OR971971 |
| LHD123 | Guatemala | Chimaltenango | *Pisum sativum* var. *saccharatum* | Inv Hap-A N245 | OR971972 |
| LHD124 | Guatemala | Chimaltenango | *Pisum sativum* var. *saccharatum* | Inv Hap-B N18 | OR971973 |
| LHD125 | Guatemala | Chimaltenango | *Pisum sativum* var. *saccharatum* | Inv Hap-A N245 | OR971974 |
| LHD126 | Guatemala | Chimaltenango | *Pisum sativum* var. *saccharatum* | Inv Hap-A N245 | OR971975 |
| LHD127 | Guatemala | Chimaltenango | *Pisum sativum* var. *saccharatum* | Inv Hap-A N245 | OR971976 |
| LHD128 | Guatemala | Chimaltenango | *Pisum sativum* var. *saccharatum* | Inv Hap-A N245 | OR971977 |
| LHD129 | Guatemala | Chimaltenango | *Pisum sativum* var. *saccharatum* | Inv Hap-A N245 | OR971978 |
| LHD130 | Guatemala | Chimaltenango | *Pisum sativum* var. *saccharatum* | Inv Hap-A N245 | OR971979 |
| LHD132 | Guatemala | Chimaltenango | *Pisum sativum* var. *saccharatum* | Inv Hap-A N245 | OR971980 |
| LHD133 | Guatemala | Chimaltenango | *Pisum sativum* var. *saccharatum* | Inv Hap-A N245 | OR971981 |
| LHD135 | Guatemala | Chimaltenango | *Pisum sativum* var. *saccharatum* | Inv Hap-A N245 | OR971982 |
| LHD136 | Guatemala | Chimaltenango | *Pisum sativum* var. *saccharatum* | Inv Hap-A N245 | OR971983 |
| LHD137 | Ecuador | Carchi | *Solanum tuberosum* | ECU Shared-1 N26 | OR971984 |
| LHD138 | Ecuador | Carchi | *Solanum tuberosum* | ECU Lhd-138 Single | OR971985 |
| LHD139 | Ecuador | Carchi | *Solanum tuberosum* | ECU Shared-1 N26 | OR971986 |
| LHD140 | Ecuador | Carchi | *Solanum tuberosum* | ECU Shared-1 N26 | OR971987 |
| LHD141 | Ecuador | Carchi | *Solanum tuberosum* | ECU Shared-1 N26 | OR971988 |
| LHD142 | Ecuador | Carchi | *Solanum tuberosum* | ECU Shared-1 N26 | OR971989 |
| LHD143 | Ecuador | Carchi | *Solanum tuberosum* | ECU Shared-2 N2 | OR971990 |
| LHD144 | Argentina | Cordoba | *Beta vulgaris* | ARG Shared-3 N2 | OR971991 |
| LHD145 | Argentina | Cordoba | *Beta vulgaris* | ARG Tlh-145 Single | OR971992 |
| LHD146 | Argentina | Cordoba | *Beta vulgaris* | ARG Shared-2 N2 | OR971993 |
| LHD147 | Costa Rica | Coronado | *Brassica oleracea* | Inv Hap-A N245 | OR971994 |
| LHD148 | Costa Rica | Coronado | *Brassica oleracea* | Inv Hap-A N245 | OR971995 |
| LHD149 | Costa Rica | Coronado | *Brassica oleracea* | Inv Hap-A N245 | OR971996 |
| LHD150 | Costa Rica | Coronado | *Brassica oleracea* | Inv Hap-A N245 | OR971997 |
| LHD151 | Costa Rica | Poas Alajuela | *Eruca vesicaria* | Inv Hap-A N245 | OR971998 |
| LHD152 | Costa Rica | Poas Alajuela | *Eruca vesicaria* | Inv Hap-A N245 | OR971999 |
| LHD153 | Costa Rica | Poas Alajuela | *Eruca vesicaria* | Inv Hap-A N245 | OR972000 |
| LHD154 | Costa Rica | Poas Alajuela | *Eruca vesicaria* | Inv Hap-A N245 | OR972001 |
| LHD155 | Costa Rica | Poas Alajuela | *Eruca vesicaria* | Inv Hap-A N245 | OR972002 |
| LHD156 | Costa Rica | Poas Alajuela | *Eruca vesicaria* | CR Lhd-156 Single | OR972003 |
| LHD157 | Costa Rica | Poas Alajuela | *Eruca vesicaria* | Inv Hap-A N245 | OR972004 |
| LHD158 | Costa Rica | Poas Alajuela | *Eruca vesicaria* | Inv Hap-A N245 | OR972005 |
| LHD159 | Costa Rica | Poas Alajuela | *Eruca vesicaria* | Inv Hap-A N245 | OR972006 |
| LHD160 | Costa Rica | Poas Alajuela | *Eruca vesicaria* | Inv Hap-A N245 | OR972007 |
| LHD161 | Costa Rica | Poas Alajuela | *Eruca vesicaria* | Inv Hap-A N245 | OR972008 |
| LHD162 | Costa Rica | Poas Alajuela | *Eruca vesicaria* | CR Lhd-162 Single | OR972009 |
| LHD163 | Costa Rica | Poas Alajuela | *Eruca vesicaria* | Inv Hap-A N245 | OR972010 |
| LHD164 | Panama | Cero Punta | *Brassica rapa* | Inv Hap-A N245 | OR972011 |
| LHD165 | Panama | Cero Punta | *Brassica rapa* | Inv Hap-A N245 | OR972012 |
| LHD166 | Panama | Cero Punta | *Brassica rapa* | Inv Hap-A N245 | OR972013 |
| LHD167 | Panama | Cero Punta | *Brassica rapa* | Inv Hap-A N245 | OR972014 |
| LHD168 | Panama | Cero Punta | *Brassica rapa* | Inv Hap-A N245 | OR972015 |
| LHD169 | Panama | Cero Punta | *Brassica rapa* | Inv Hap-A N245 | OR972016 |
| LHD170 | Panama | Cero Punta | *Brassica rapa* | Inv Hap-A N245 | OR972017 |
| LHD172 | Panama | Cero Punta | *Brassica rapa* | PAN Lhd-172 Single | OR972018 |
| LHD173 | Colombia | Pradera | *Phaseolus vulgaris* | COL Shared-1 N19 | OR972019 |
| LHD174 | Colombia | Pradera | *Phaseolus vulgaris* | COL Shared-1 N19 | OR972020 |
| LHD175 | Colombia | Pradera | *Phaseolus vulgaris* | COL Shared-1 N19 | OR972021 |
| LHD176 | Colombia | Pradera | *Phaseolus vulgaris* | COL Shared-1 N19 | OR972022 |
| LHD177 | Colombia | Pradera | *Phaseolus vulgaris* | COL Shared-1 N19 | OR972023 |
| LHD178 | Colombia | Pradera | *Phaseolus vulgaris* | COL Shared-1 N19 | OR972024 |
| LHD179 | Colombia | Pradera | *Phaseolus vulgaris* | COL Shared-1 N19 | OR972025 |
| LHD180 | Colombia | Pradera | *Phaseolus vulgaris* | COL Shared-1 N19 | OR972026 |
| LHD181 | Colombia | Pradera | *Phaseolus vulgaris* | COL Shared-1 N19 | OR972027 |
| LHD182 | Colombia | Pradera | *Phaseolus vulgaris* | COL Shared-1 N19 | OR972028 |
| LHD183 | Colombia | Pradera | *Phaseolus vulgaris* | COL Lhd-183 Single | OR972029 |
| LHD184 | Colombia | Pradera | *Phaseolus vulgaris* | COL Shared-1 N19 | OR972030 |
| LHD185 | Colombia | Pradera | *Phaseolus vulgaris* | COL Lhd-185 Single | OR972031 |
| LHD186 | Colombia | Pradera | *Phaseolus vulgaris* | COL Shared-1 N19 | OR972032 |
| LHD187 | Colombia | Pradera | *Phaseolus vulgaris* | COL Shared-1 N19 | OR972033 |
| LHD188 | Colombia | Pradera | *Phaseolus vulgaris* | COL Shared-1 N19 | OR972034 |
| LHD189 | Colombia | Pradera | *Phaseolus vulgaris* | COL Shared-1 N19 | OR972035 |
| LHD190 | Colombia | Pradera | *Phaseolus vulgaris* | COL Shared-1 N19 | OR972036 |
| LHD191 | Peru | Lima, San Agustin | *Pisum sativum* | Inv Hap-A N245 | OR972037 |
| LHD193 | Peru | Lima, San Agustin | *Pisum sativum* | Inv Hap-A N245 | OR972038 |
| LHD194 | Peru | Lima, San Agustin | *Pisum sativum* | Inv Hap-A N245 | OR972039 |
| LHD195 | Peru | Lima, San Agustin | *Pisum sativum* | Inv Hap-A N245 | OR972040 |
| LHD196 | Peru | Lima, San Agustin | *Pisum sativum* | Inv Hap-A N245 | OR972041 |
| LHD197 | Peru | Lima, San Agustin | *Pisum sativum* | Inv Hap-A N245 | OR972042 |
| LHD198 | Peru | Lima, San Agustin | *Pisum sativum* | Inv Hap-A N245 | OR972043 |
| LHD199 | Peru | Lima, San Agustin | *Pisum sativum* | Inv Hap-A N245 | OR972044 |
| LHD200 | Peru | Lima, San Agustin | *Pisum sativum* | Inv Hap-A N245 | OR972045 |
| LHD201 | Peru | La Libertad | *Vicia faba* | Inv Hap-C N2 | OR972046 |
| LHD202 | Peru | La Libertad | *Vicia faba* | PE Shared-1 N13 | OR972047 |
| LHD203 | Peru | La Libertad | *Vicia faba* | PE Lhd-203 Single | OR972048 |
| LHD204 | Peru | La Libertad | *Vicia faba* | PE Shared-1 N13 | OR972049 |
| LHD206 | Peru | La Libertad | *Vicia faba* | PE Shared-2 N26 | OR972050 |
| LHD207 | Peru | La Libertad | *Vicia faba* | PE Shared-2 N26 | OR972051 |
| LHD208 | Peru | La Libertad | *Vicia faba* | PE Shared-2 N26 | OR972052 |
| LHD209 | Peru | La Libertad | *Vicia faba* | Inv Hap-B N18 | OR972053 |
| LHD210 | Peru | La Libertad | *Vicia faba* | PE Shared-2 N26 | OR972054 |
| LHD211 | Peru | Lima, La Molina | *Solanum tuberosum* | Inv Hap-D N2 | OR972055 |
| LHD212 | Peru | Lima, La Molina | *Solanum tuberosum* | Inv Hap-A N245 | OR972056 |
| LHD213 | Peru | Lima, La Molina | *Solanum tuberosum* | PE Lhd-213 Single | OR972057 |
| LHD214 | Peru | Lima, La Molina | *Solanum tuberosum* | Inv Hap-A N245 | OR972058 |
| LHD215 | Peru | Lima, La Molina | *Solanum tuberosum* | PE Lhd-215 Single | OR972059 |
| LHD216 | Peru | Lima, La Molina | *Solanum tuberosum* | Inv Hap-A N245 | OR972060 |
| LHD217 | Peru | Lima, La Molina | *Solanum tuberosum* | Inv Hap-A N245 | OR972061 |
| LHD218 | Peru | Lima, La Molina | *Solanum tuberosum* | Inv Hap-A N245 | OR972062 |
| LHD220 | Peru | Lima, La Molina | *Solanum tuberosum* | Inv Hap-A N245 | OR972063 |
| LHD231 | Ecuador | Chimborazo | *Solanum tuberosum* | ECU Lhd231 single | OR972064 |
| LHD232 | Ecuador | Chimborazo | *Solanum tuberosum* | ECU Shared-1 N26 | OR972065 |
| LHD233 | Ecuador | Chimborazo | *Solanum tuberosum* | ECU Shared-3 N3 | OR972066 |
| LHD236 | Ecuador | Chimborazo | *Solanum tuberosum* | ECU Lhd236 Single | OR972067 |
| LHD237 | Ecuador | Chimborazo | *Solanum tuberosum* | ECU Shared-3 N3 | OR972068 |
| LHD238 | Ecuador | Chimborazo | *Solanum tuberosum* | ECU Shared-3 N3 | OR972069 |
| LHD239 | Ecuador | Chimborazo | *Solanum tuberosum* | Inv Hap-A N245 | OR972070 |
| LHD240 | Ecuador | Chimborazo | *Solanum tuberosum* | ECU Lhd240 Single | OR972071 |
| LHD241 | Ecuador | Carchi | *Solanum tuberosum* | ECU Shared-5 N5 | OR972072 |
| LHD242 | Ecuador | Carchi | *Solanum tuberosum* | Inv Hap-A N245 | OR972073 |
| LHD243 | Ecuador | Carchi | *Solanum tuberosum* | ECU Shared-1 N26 | OR972074 |
| LHD244 | Ecuador | Carchi | *Solanum tuberosum* | ECU Shared-1 N26 | OR972075 |
| LHD245 | Ecuador | Carchi | *Solanum tuberosum* | ECU Shared-5 N5 | OR972076 |
| LHD246 | Ecuador | Carchi | *Solanum tuberosum* | ECU Shared-1 N26 | OR972077 |
| LHD247 | Ecuador | Carchi | *Solanum tuberosum* | ECU Shared-2 N2 | OR972078 |
| LHD248 | Ecuador | Carchi | *Solanum tuberosum* | ECU Shared-5 N5 | OR972079 |
| LHD269 | South Africa | Sanvelt Region | *Solanum tuberosum* | Inv Hap-A N245 | OR972080 |
| LHD270 | South Africa | Sanvelt Region | *Solanum tuberosum* | Inv Hap-A N245 | OR972081 |
| LHD271 | South Africa | Sanvelt Region | *Solanum tuberosum* | Inv Hap-A N245 | OR972082 |
| LHD272 | South Africa | Sanvelt Region | *Solanum tuberosum* | Inv Hap-A N245 | OR972083 |
| LHD273 | South Africa | Sanvelt Region | *Solanum tuberosum* | Inv Hap-A N245 | OR972084 |
| LHD274 | South Africa | Sanvelt Region | *Solanum tuberosum* | Inv Hap-A N245 | OR972085 |
| LHD275 | South Africa | Sanvelt Region | *Solanum tuberosum* | Inv Hap-A N245 | OR972086 |
| LHD276 | South Africa | Sanvelt Region | *Solanum tuberosum* | Inv Hap-A N245 | OR972087 |
| LHD278 | South Africa | Sanvelt Region | *Solanum tuberosum* | Inv Hap-A N245 | OR972088 |
| LHD279 | South Africa | Sanvelt Region | *Solanum tuberosum* | Inv Hap-A N245 | OR972089 |
| LHD280 | South Africa | Sanvelt Region | *Solanum tuberosum* | Inv Hap-A N245 | OR972090 |
| LHD281 | South Africa | Sanvelt Region | *Solanum tuberosum* | Inv Hap-A N245 | OR972091 |
| LHD282 | South Africa | Sanvelt Region | *Solanum tuberosum* | Inv Hap-A N245 | OR972092 |
| LHD283 | South Africa | Sanvelt Region | *Solanum tuberosum* | Inv Hap-A N245 | OR972093 |
| LHD284 | South Africa | Sanvelt Region | *Solanum tuberosum* | Inv Hap-A N245 | OR972094 |
| LHD286 | South Africa | Sanvelt Region | *Solanum tuberosum* | Inv Hap-A N245 | OR972095 |
| LHD287 | South Africa | Sanvelt Region | *Solanum tuberosum* | Inv Hap-A N245 | OR972096 |
| LHD288 | South Africa | Sanvelt Region | *Solanum tuberosum* | Inv Hap-A N245 | OR972097 |
| LHD289 | South Africa | Sanvelt Region | *Solanum tuberosum* | Inv Hap-A N245 | OR972098 |
| LHD290 | Ecuador | Pichincha | *Gypsophia* sp. | ECU Shared-1 N26 | OR972099 |
| LHD291 | Ecuador | Pichincha | *Gypsophia* sp. | ECU Shared-1 N26 | OR972100 |
| LHD292 | Ecuador | Pichincha | *Gypsophia* sp. | ECU Shared-1 N26 | OR972101 |
| LHD293 | Ecuador | Pichincha | *Gypsophia* sp. | ECU Shared-1 N26 | OR972102 |
| LHD294 | Ecuador | Pichincha | *Gypsophia* sp. | ECU Shared-1 N26 | OR972103 |
| LHD295 | Ecuador | Pichincha | *Gypsophia* sp. | ECU Shared-1 N26 | OR972104 |
| LHD296 | Ecuador | Pichincha | *Gypsophia* sp. | ECU Lhd-296 Single | OR972105 |
| LHD297 | Ecuador | Pichincha | *Gypsophia* sp. | ECU Shared-1 N26 | OR972106 |
| LHD298 | Ecuador | Pichincha | *Gypsophia* sp. | ECU Shared-1 N26 | OR972107 |
| LHD299 | Ecuador | Pichincha | *Gypsophia* sp. | ECU Lhd-299 Single | OR972108 |
| LHD300 | Ecuador | Pichincha | *Gypsophia* sp. | ECU Shared-1 N26 | OR972109 |
| LHD301 | Ecuador | Pichincha | *Gypsophia* sp. | ECU Shared-1 N26 | OR972110 |
| LHD302 | Ecuador | Pichincha | *Gypsophia* sp. | ECU Shared-1 N26 | OR972111 |
| LHD303 | Ecuador | Pichincha | *Gypsophia* sp. | ECU Shared-1 N26 | OR972112 |
| LHD304 | Ecuador | Pichincha | *Gypsophia* sp. | ECU Lhd-304 Single | OR972113 |
| LHD305 | Ecuador | Pichincha | *Gypsophia* sp. | ECU Shared-1 N26 | OR972114 |
| LHD306 | Ecuador | Pichincha | *Gypsophia* sp. | ECU Shared-1 N26 | OR972115 |
| LHD307 | Ecuador | Pichincha | *Gypsophia* sp. | ECU Shared-1 N26 | OR972116 |
| LHD308 | Ecuador | Pichincha | *Gypsophia* sp. | ECU Shared-1 N26 | OR972117 |
| LHD309 | Ecuador | Pichincha | *Gypsophia* sp. | ECU Shared-1 N26 | OR972118 |
| LHD310 | Indonesia | Java | *Solanum tuberosum* | Inv Hap-B N18 | OR972119 |
| LHD311 | Indonesia | Java | *Solanum tuberosum* | Inv Hap-A N245 | OR972120 |
| LHD312 | Indonesia | Java | *Solanum tuberosum* | Inv Hap-A N245 | OR972121 |
| LHD313 | Indonesia | Java | *Solanum tuberosum* | Inv Hap-A N245 | OR972122 |
| LHD314 | Indonesia | Java | *Solanum tuberosum* | Inv Hap-A N245 | OR972123 |
| LHD315 | Indonesia | Java | *Solanum tuberosum* | Inv Hap-B N18 | OR972124 |
| LHD316 | Indonesia | Java | *Solanum tuberosum* | IND only N5 | OR972125 |
| LHD317 | Indonesia | Java | *Solanum tuberosum* | IND only N5 | OR972126 |
| LHD318 | Indonesia | Java | *Solanum tuberosum* | Inv Hap-A N245 | OR972127 |
| LHD319 | Indonesia | Java | *Solanum tuberosum* | IND only N5 | OR972128 |
| LHD320 | Indonesia | Java | *Solanum tuberosum* | Inv Hap-A N245 | OR972129 |
| LHD321 | Indonesia | Java | *Solanum tuberosum* | IND only N5 | OR972130 |
| LHD322 | Indonesia | Java | *Solanum tuberosum* | Inv Hap-B N18 | OR972131 |
| LHD323 | Indonesia | Java | *Solanum tuberosum* | Inv Hap-A N245 | OR972132 |
| LHD324 | China | Bejing | lab colony | Inv Hap-A N245 | OR972133 |
| LHD325 | China | Bejing | lab colony | Inv Hap-A N245 | OR972134 |
| LHD326 | China | Bejing | lab colony | Inv Hap-A N245 | OR972135 |
| LHD327 | China | Bejing | lab colony | Inv Hap-A N245 | OR972136 |
| LHD328 | China | Bejing | lab colony | Inv Hap-A N245 | OR972137 |
| LHD329 | China | Bejing | lab colony | Inv Hap-A N245 | OR972138 |
| LHD330 | China | Bejing | lab colony | Inv Hap-A N245 | OR972139 |
| LHD331 | China | Bejing | lab colony | Inv Hap-A N245 | OR972140 |
| LHD332 | Italy | Bologna/Cesena | lab colony | Inv Hap-A N245 | OR972141 |
| LHD333 | Italy | Bologna/Cesena | lab colony | Inv Hap-A N245 | OR972142 |
| LHD334 | Italy | Bologna/Cesena | lab colony | Inv Hap-A N245 | OR972143 |
| LHD335 | Italy | Bologna/Cesena | lab colony | Inv Hap-A N245 | OR972144 |
| LHD336 | Italy | Bologna/Cesena | lab colony | Inv Hap-A N245 | OR972145 |
| LHD337 | Italy | Bologna/Cesena | lab colony | Inv Hap-A N245 | OR972146 |
| LHD338 | Italy | Bologna/Cesena | lab colony | Inv Hap-B N18 | OR972147 |
| LHD339 | Italy | Bologna/Cesena | lab colony | Inv Hap-A N245 | OR972148 |
| LHD340 | Colombia | Pradera | *Phaseolus vulgaris* | COL Lhd-340 Single | OR972149 |
| LHD343 | Colombia | Pradera | *Phaseolus vulgaris* | COL Shared-1 N19 | OR972150 |
| LHD349 | Israel | Gilat Experimental Station | *Solanum tuberosum* | Inv Hap-B N18 | OR972151 |
| LHD350 | Israel | Gilat Experimental Station | *Solanum tuberosum* | Inv Hap-A N245 | OR972152 |
| LHD351 | Israel | Gilat Experimental Station | *Solanum tuberosum* | Inv Hap-A N245 | OR972153 |
| LHD352 | Israel | Gilat Experimental Station | *Solanum tuberosum* | ISR Lhd-352 Single | OR972154 |
| LHD353 | Israel | Gilat Experimental Station | *Solanum tuberosum* | Inv Hap-A N245 | OR972155 |
| LHD354 | Israel | Gilat Experimental Station | *Solanum tuberosum* | Inv Hap-A N245 | OR972156 |
| LHD355 | Israel | Gilat Experimental Station | *Solanum tuberosum* | Inv Hap-A N245 | OR972157 |
| LHD356 | Israel | Gilat Experimental Station | *Solanum tuberosum* | Inv Hap-A N245 | OR972158 |
| LHD357 | Israel | Gilat Experimental Station | *Solanum tuberosum* | Inv Hap-A N245 | OR972159 |
| LPPQ102 | Netherlands | USDA-APHIS PPQ | *Ranunculus* sp. | Inv Hap-A N245 | OR972164 |
| LPPQ103 | Kenya | USDA-APHIS PPQ | *Ranunculus* sp. | Inv Hap-A N245 | OR972165 |
| LPPQ104 | Kenya | USDA-APHIS PPQ | *Ranunculus* sp. | Inv Hap-A N245 | OR972166 |
| LPPQ106 | Netherlands | USDA-APHIS PPQ | *Ranunculus* sp. | Inv Hap-A N245 | OR972167 |
| LPPQ107 | Netherlands | USDA-APHIS PPQ | *Ranunculus* sp. | NETH LPPQ-107 single | OR972168 |
| LPPQ108 | Colombia | USDA-APHIS PPQ | *Artemisia* sp. | PPQ Shared-1 N3 | OR972169 |
| LPPQ50 | Colombia | USDA-APHIS PPQ | *Artemisia* sp. | COL LPPQ-50 single | OR972160 |
| LPPQ85 | Netherlands | USDA-APHIS PPQ | *Ranunculus* sp. | Inv Hap-A N245 | OR972161 |
| LPPQ86 | Netherlands | USDA-APHIS PPQ | *Ranunculus* sp. | Inv Hap-A N245 | OR972162 |
| LPPQ88 | Netherlands | USDA-APHIS PPQ | *Ranunculus* sp. | Inv Hap-A N245 | OR972163 |
| RJ10 | Philippines | Banaue, Ifugao Province | *Brassica rapa* | Inv Hap-A N245 | OR972170 |
| RJ100 | Philippines | Mountain Province | beans | Inv Hap-A N245 | OR972200 |
| RJ101 | Philippines | Benguet Province | *Brassica rapa* | Inv Hap-A N245 | OR972201 |
| RJ103 | Philippines | Benguet Province | *Brassica rapa* | Inv Hap-A N245 | OR972202 |
| RJ107 | Philippines | Benguet Province | *Apium graveolens* | Inv Hap-A N245 | OR972203 |
| RJ108 | Philippines | Benguet Province | *Apium graveolens* | Inv Hap-A N245 | OR972204 |
| RJ110 | Philippines | Benguet Province | *Allium cepa* | Inv Hap-A N245 | OR972205 |
| RJ111 | Philippines | Benguet Province | *Allium cepa* | Inv Hap-A N245 | OR972206 |
| RJ112 | Philippines | Benguet Province | *Allium cepa* | Inv Hap-A N245 | OR972207 |
| RJ113 | Philippines | Benguet Province | *Allium cepa* | Inv Hap-A N245 | OR972208 |
| RJ114 | Philippines | Mountain Province | sida | Inv Hap-A N245 | OR972209 |
| RJ124 | Philippines | Banaue, Ifugao Province | beans | Inv Hap-A N245 | OR972210 |
| RJ134 | Philippines | Banaue, Ifugao Province | sida | Inv Hap-A N245 | OR972211 |
| RJ138 | Philippines | Banaue, Ifugao Province | *Brassica oleracea* | Inv Hap-A N245 | OR972212 |
| RJ139 | Philippines | Banaue, Ifugao Province | *Brassica oleracea* | Inv Hap-A N245 | OR972213 |
| RJ14 | Philippines | Banaue, Ifugao Province | peas | Inv Hap-A N245 | OR972171 |
| RJ140 | Philippines | Banaue, Ifugao Province | *Brassica oleracea* | Inv Hap-A N245 | OR972214 |
| RJ141 | Philippines | Banaue, Ifugao Province | *Brassica oleracea* | Inv Hap-A N245 | OR972215 |
| RJ144 | Philippines | Banaue, Ifugao Province | *Brassica rapa ssp. chinensis* | Inv Hap-A N245 | OR972216 |
| RJ148 | Philippines | Banaue, Ifugao Province | beans | Inv Hap-A N245 | OR972217 |
| RJ15 | Philippines | Banaue, Ifugao Province | peas | Inv Hap-A N245 | OR972172 |
| RJ16 | Philippines | Banaue, Ifugao Province | peas | Inv Hap-A N245 | OR972173 |
| RJ212 | Philippines | Mountain Province | zucchini | Inv Hap-A N245 | OR972218 |
| RJ213 | Philippines | Mountain Province | zucchini | Inv Hap-A N245 | OR972219 |
| RJ222 | Philippines | Mountain Province | *Solanum lycopersicum* | Inv Hap-A N245 | OR972220 |
| RJ23 | Philippines | Banaue, Ifugao Province | *Brassica rapa ssp. chinensis* | Inv Hap-A N245 | OR972174 |
| RJ246 | Philippines | Benguet Province | dangey | Inv Hap-A N245 | OR972221 |
| RJ248 | Philippines | Benguet Province | *Sonchus oleraceus* | Inv Hap-A N245 | OR972222 |
| RJ249 | Philippines | Benguet Province | *Sonchus oleraceus* | Inv Hap-A N245 | OR972223 |
| RJ253 | Philippines | Benguet Province | *Lactuca sativa* | Inv Hap-A N245 | OR972224 |
| RJ254 | Philippines | Benguet Province | *Lactuca sativa* | Inv Hap-A N245 | OR972225 |
| RJ27 | Philippines | Benguet Province | beans | Inv Hap-A N245 | OR972175 |
| RJ28 | Philippines | Benguet Province | beans | Inv Hap-A N245 | OR972176 |
| RJ29 | Philippines | Benguet Province | *Brassica oleracea* | Inv Hap-A N245 | OR972177 |
| RJ30 | Philippines | Benguet Province | *Brassica oleracea* | Inv Hap-A N245 | OR972178 |
| RJ31 | Philippines | Benguet Province | *Brassica oleracea* | Inv Hap-A N245 | OR972179 |
| RJ32 | Philippines | Benguet Province | *Brassica oleracea* | Inv Hap-A N245 | OR972180 |
| RJ34 | Philippines | Benguet Province | carrots | Inv Hap-A N245 | OR972181 |
| RJ36 | Philippines | Benguet Province | carrots | Inv Hap-A N245 | OR972182 |
| RJ37 | Philippines | Benguet Province | *Apium graveolens* | Inv Hap-A N245 | OR972183 |
| RJ38 | Philippines | Benguet Province | *Apium graveolens* | Inv Hap-A N245 | OR972184 |
| RJ39 | Philippines | Benguet Province | *Apium graveolens* | Inv Hap-A N245 | OR972185 |
| RJ43 | Philippines | Benguet Province | *Chrysanthemum* sp. | Inv Hap-A N245 | OR972186 |
| RJ49 | Philippines | Benguet Province | *Arctium lappa* | Inv Hap-A N245 | OR972187 |
| RJ50 | Philippines | Benguet Province | *Arctium lappa* | Inv Hap-A N245 | OR972188 |
| RJ69 | Philippines | Balili | beans | Inv Hap-A N245 | OR972189 |
| RJ71 | Philippines | Balili | beans | Inv Hap-A N245 | OR972190 |
| RJ72 | Philippines | Balili | beans | Inv Hap-A N245 | OR972191 |
| RJ75 | Philippines | Benguet Province | *Solanum lycopersicum* | Inv Hap-A N245 | OR972192 |
| RJ85 | Philippines | Benguet Province | *Solanum lycopersicum* | Inv Hap-A N245 | OR972193 |
| RJ86 | Philippines | Benguet Province | *Solanum lycopersicum* | Inv Hap-A N245 | OR972194 |
| RJ93 | Philippines | Benguet Province | beans | Inv Hap-A N245 | OR972195 |
| RJ94 | Philippines | Benguet Province | beans | Inv Hap-A N245 | OR972196 |
| RJ97 | Philippines | Mountain Province | beans | Inv Hap-A N245 | OR972197 |
| RJ98 | Philippines | Mountain Province | beans | Inv Hap-A N245 | OR972198 |
| RJ99 | Philippines | Mountain Province | beans | Inv Hap-A N245 | OR972199 |
| TLH105 | Sri Lanka | Galpalama | *Allium ampeloprasum* | Inv Hap-A N245 | OR972282 |
| TLH106 | Sri Lanka | Galpalama | *Brassica oleracea* | Inv Hap-A N245 | OR972283 |
| TLH107 | Sri Lanka | Galpalama | *Brassica oleracea* | Inv Hap-A N245 | OR972284 |
| TLH109 | South Africa | Sanvelt Region | *Solanum tuberosum* | Inv Hap-A N245 | OR972285 |
| TLH11 | Guatemala | Chimaltenango | *Vicia faba* | Inv Hap-A N245 | OR972228 |
| TLH110 | South Africa | Sanvelt Region | *Solanum tuberosum* | Inv Hap-A N245 | OR972286 |
| TLH112 | South Africa | Sanvelt Region | *Solanum tuberosum* | Inv Hap-A N245 | OR972287 |
| TLH113 | South Africa | Sanvelt Region | *Solanum tuberosum* | Inv Hap-A N245 | OR972288 |
| TLH114 | South Africa | Sanvelt Region | *Solanum tuberosum* | Inv Hap-A N245 | OR972289 |
| TLH115 | South Africa | Sanvelt Region | *Solanum tuberosum* | Inv Hap-A N245 | OR972290 |
| TLH117 | South Africa | Sanvelt Region | *Solanum tuberosum* | Inv Hap-A N245 | OR972291 |
| TLH119 | Canada | Ontario | near greenhouses | Inv Hap-B N18 | OR972292 |
| TLH12 | Guatemala | Chimaltenango | *Vicia faba* | Inv Hap-A N245 | OR972229 |
| TLH122 | Canada | Ontario | near greenhouses | Inv Hap-A N245 | OR972293 |
| TLH124 | Sri Lanka | Sita Eliya | *Brassica rapa* | Inv Hap-A N245 | OR972294 |
| TLH125 | Sri Lanka | Sita Eliya | *Brassica rapa* | Inv Hap-A N245 | OR972295 |
| TLH126 | Sri Lanka | Galpalama | *Beta vulgaris* | Inv Hap-A N245 | OR972296 |
| TLH127 | Sri Lanka | Galpalama | *Beta vulgaris* | Inv Hap-A N245 | OR972297 |
| TLH128 | Sri Lanka | Sita Eliya | emilia | Inv Hap-A N245 | OR972298 |
| TLH13 | Guatemala | Chimaltenango | weed | Inv Hap-A N245 | OR972230 |
| TLH134 | Colombia | Pradera | *Phaseolus vulgaris* | COL Shared-1 N19 | OR972299 |
| TLH135 | Colombia | Pradera | *Phaseolus vulgaris* | COL Shared-1 N19 | OR972300 |
| TLH136 | Guatemala | Chimaltenango | *Pisum sativum* var. *saccharatum* | Inv Hap-A N245 | OR972301 |
| TLH137 | Guatemala | Chimaltenango | *Pisum sativum* var. *saccharatum* | GUA Tlh-137 Single | OR972302 |
| TLH14 | Sri Lanka | Sita Eliya | *Chrysanthemum* sp. | Inv Hap-A N245 | OR972231 |
| TLH140 | Peru | Cañete | *Medicago sativa* | Inv Hap-A N245 | OR972303 |
| TLH141 | Peru | Cañete | *Medicago sativa* | Inv Hap-A N245 | OR972304 |
| TLH143 | China | Bejing | colony | Inv Hap-A N245 | OR972305 |
| TLH146 | Peru | Lima, La Molina | *Solanum tuberosum* | Inv Hap-A N245 | OR972306 |
| TLH147 | Peru | Lima, La Molina | *Solanum tuberosum* | Inv Hap-A N245 | OR972307 |
| TLH15 | Sri Lanka | Galpalama | *Allium ampeloprasum* | Inv Hap-A N245 | OR972232 |
| TLH150 | Colombia | USDA-APHIS PPQ | *Chrysanthemum* sp. | PPQ Shared-1 N3 | OR972308 |
| TLH151 | Colombia | USDA-APHIS PPQ | "aster" | COL Tlh-151 Single | OR972309 |
| TLH152 | Ecuador | USDA-APHIS PPQ | eustoma | ECU Shared-2 N2 | OR972310 |
| TLH153 | Ecuador | USDA-APHIS PPQ | *Gypsophia* sp. | ECU Shared-1 N26 | OR972311 |
| TLH16 | Sri Lanka | Galpalama | *Brassica rapa* | Inv Hap-A N245 | OR972233 |
| TLH17 | Sri Lanka | Sita Eliya | brassica | Inv Hap-A N245 | OR972234 |
| TLH170 | Ecuador | Carchi | *Solanum tuberosum* | Inv Hap-A N245 | OR972312 |
| TLH171 | Ecuador | Carchi | *Solanum tuberosum* | ECU Shared-5 N5 | OR972313 |
| TLH172 | Ecuador | Carchi | *Solanum tuberosum* | Inv Hap-A N245 | OR972314 |
| TLH173 | Ecuador | Carchi | *Solanum tuberosum* | ECU Shared-5 N5 | OR972315 |
| TLH174 | Argentina | Cordoba | *Beta vulgaris* | ARG Shared-1 N3 | OR972316 |
| TLH175 | Argentina | Cordoba | *Beta vulgaris* | ARG Shared-1 N3 | OR972317 |
| TLH176 | Argentina | Cordoba | *Beta vulgaris* | ARG Tlh-176 Single | OR972318 |
| TLH177 | Argentina | Cordoba | *Beta vulgaris* | ARG Shared-2 N2 | OR972319 |
| TLH18 | Israel | Gilat Experimental Station | *Solanum tuberosum* | Inv Hap-A N245 | OR972235 |
| TLH182 | Peru | Lima, La Molina | *Solanum tuberosum* | Inv Hap-A N245 | OR972320 |
| TLH183 | Peru | Lima, La Molina | *Solanum tuberosum* | PE Tlh-183 Single | OR972321 |
| TLH184 | Peru | Lima, La Molina | *Solanum tuberosum* | Inv Hap-A N245 | OR972322 |
| TLH185 | Peru | Lima, La Molina | *Solanum tuberosum* | Inv Hap-A N245 | OR972323 |
| TLH186 | Guatemala | Chimaltenango | *Pisum sativum* var. *saccharatum* | Inv Hap-A N245 | OR972324 |
| TLH187 | Guatemala | Chimaltenango | *Pisum sativum* var. *saccharatum* | Inv Hap-A N245 | OR972325 |
| TLH19 | Israel | Gilat Experimental Station | *Solanum tuberosum* | Inv Hap-D N2 | OR972236 |
| TLH20 | Malaysia | Pahang | *Solanum lycopersicum* | Inv Hap-A N245 | OR972237 |
| TLH21 | Malaysia | Pahang | *Capsicum annuum* | Inv Hap-A N245 | OR972238 |
| TLH22 | Israel | Gilat Experimental Station | *Apium graveolens* | Inv Hap-C N2 | OR972239 |
| TLH23 | Israel | Gilat Experimental Station | *Lactuca sativa* | Inv Hap-A N245 | OR972240 |
| TLH24 | Indonesia | Java | *Solanum tuberosum* | IND only N5 | OR972241 |
| TLH25 | Indonesia | Java | *Solanum tuberosum* | Inv Hap-B N18 | OR972242 |
| TLH26 | Indonesia | Garat | *Solanum tuberosum* | Inv Hap-A N245 | OR972243 |
| TLH30 | Ecuador | Carchi | *Solanum tuberosum* | ECU Tlh30 Single | OR972244 |
| TLH31 | Ecuador | Carchi | *Solanum tuberosum* | ECU Tlh31 Single | OR972245 |
| TLH34 | Sri Lanka | Galpalama | *Beta vulgaris* | Inv Hap-A N245 | OR972246 |
| TLH35 | Sri Lanka | Sita Eliya | emilia | Inv Hap-A N245 | OR972247 |
| TLH4 | Guatemala | Chimaltenango | *Pisum sativum* var. *saccharatum* | Inv Hap-A N245 | OR972226 |
| TLH42 | South Africa | Sanvelt Region | *Solanum tuberosum* | Inv Hap-A N245 | OR972248 |
| TLH43 | South Africa | Sanvelt Region | *Solanum tuberosum* | Inv Hap-A N245 | OR972249 |
| TLH46 | Peru | Huanacayo | *Vicia faba* | PE Shared-2 N26 | OR972250 |
| TLH47 | Peru | Huanacayo | *Vicia faba* | PE Shared-2 N26 | OR972251 |
| TLH49 | Peru | Lima, Carabayllo | *Lactuca sativa* | PE Tlh-49 Single | OR972252 |
| TLH5 | Guatemala | Chimaltenango | *Pisum sativum* var. *saccharatum* | Inv Hap-A N245 | OR972227 |
| TLH50 | Canada | Ontario | near greenhouses | Inv Hap-A N245 | OR972253 |
| TLH51 | Canada | Ontario | near greenhouses | Inv Hap-A N245 | OR972254 |
| TLH52 | Canada | Ontario | near greenhouses | Inv Hap-A N245 | OR972255 |
| TLH54 | Italy | Bologna/Cesena | lab colony | Inv Hap-A N245 | OR972256 |
| TLH55 | Argentina | Cordoba | *Beta vulgaris* | ARG Shared-3 N2 | OR972257 |
| TLH56 | Argentina | Cordoba | *Beta vulgaris* | ARG Shared-1 N3 | OR972258 |
| TLH57 | South Africa | Vivo Region | *Solanum tuberosum* | Inv Hap-A N245 | OR972259 |
| TLH58 | South Africa | Vivo Region | *Solanum tuberosum* | Inv Hap-A N245 | OR972260 |
| TLH59 | South Africa | Vivo Region | *Solanum tuberosum* | Inv Hap-A N245 | OR972261 |
| TLH60 | Peru | Lima, Carabayllo | *Lactuca sativa* | Inv Hap-A N245 | OR972262 |
| TLH61 | Peru | Lima, San Agustin | *Solanum lycopersicum* | Inv Hap-A N245 | OR972263 |
| TLH62 | Peru | Lima, San Agustin | *Apium graveolens* | Inv Hap-A N245 | OR972264 |
| TLH63 | Peru | Lima, Carabayllo | *Phaseolus vulgaris* | Inv Hap-A N245 | OR972265 |
| TLH65 | Peru | Lima, San Agustin | *Allium cepa* | PE Shared-2 N26 | OR972266 |
| TLH66 | Peru | La Libertad | *Vicia faba* | PE Shared-1 N13 | OR972267 |
| TLH67 | Peru | Huanacayo | *Lupinus mutabilis* | Inv Hap-A N245 | OR972268 |
| TLH69 | Peru | La Libertad | *Vicia faba* | PE Shared-2 N26 | OR972269 |
| TLH70 | Peru | Lima, La Molina | *Solanum tuberosum* | Inv Hap-A N245 | OR972270 |
| TLH71 | Peru | Lima, Carabayllo | beans | Inv Hap-A N245 | OR972271 |
| TLH72 | Peru | Cañete | *Solanum tuberosum* | Inv Hap-A N245 | OR972272 |
| TLH75 | Peru | Lima, San Agustin | *Spinacia oleracea* | Inv Hap-A N245 | OR972273 |
| TLH76 | Peru | Lima, Carabayllo | *Raphanus raphanistrum* | PE Tlh-76 Single | OR972274 |
| TLH77 | Peru | Lima, Carabayllo | *Apium graveolens* | Inv Hap-A N245 | OR972275 |
| TLH79 | Philippines | Banaue, Ifugao Province | *Brassica rapa ssp. chinensis* | Inv Hap-A N245 | OR972276 |
| TLH81 | Philippines | Benguet Province | *Lactuca sativa* | Inv Hap-A N245 | OR972277 |
| TLH90 | Canada | Ontario | near greenhouses | Inv Hap-A N245 | OR972278 |
| TLH91 | Canada | Ontario | near greenhouses | Inv Hap-A N245 | OR972279 |
| TLH94 | Canada | Ontario | near greenhouses | CAN Tlh-94 Single | OR972280 |
| TLH98 | Canada | Ontario | near greenhouses | Inv Hap-A N245 | OR972281 |

Suppl Table 2. Primer sequences and directions (Forward, Reverse) used in this study.

| **Primer Name** | **Direction** | **Sequences 5'-3'** | **Reference** |
| --- | --- | --- | --- |
| TY-J-1461 | F | TTTACARTTTACCGCCTATTRTCAGCCA | Winkler et al. (2009) |
| LCO1490 | F | GGTCAACAAATCATAAAGATATTGG | Folmer et al. (1994) |
| LhdCO-1997 | F | CGATCAACTGGTATTACTTTTGATCG | this study |
| C1-J-2183 | F | CAACATTTATTTTGATTTTTTGG | Simon et al. (1994) |
| LhdCO-2191 | R | CGGGTAAAATTAAAATATAAACTTC | this study |
| HCO2198 | R | TAAACTTCAGGGTGACCAAAAAATCA | Folmer et al. (1994) |
| C1-N-2413 | R | TCARCTRAAAATTTTAATTCCTGT | Winkler et al. (2009) |
| LhdCO-2795 | F | CGACGATATTCTGATTACCCAGATGC | this study |
| LhdCO-2812 | R | GAAGATCCGATTGTGGAGATTAC | this study |
| TL2-N-3014 | R | TCCATTGCACTAATCTGCCATATTA | Simon et al. (1994) |
| LhdCO-3245 | F | CAAACCGACTATTATTACACGGAC | this study |
| LhdCO-3275 | R | GCAATAAATAAAAGAATAATAGCAGG | this study |
| C2-J-3408 | F | GTCACCAATGATAYTGAAGTTATGA | Simon et al. (1994) |
| TD-N-3862 | R | TTTAGTTTGACATACTAATGTTAT | Simon et al. (1994) |

References

Folmer, O., Black, M., Hoeh, W., Lutz, R., Vrijenhoek, R. (1994). DNA primers for amplification of mitochondrial cytochrome c oxidase subunit I from diverse metazoan invertebrates. *Molecular Marine Biology Biotechnology,* 3, 294-299.

Simon, C., Frati, F., Beckenbach, A., Crespi, B., Liu, H., Flook, P. (1994). Evolution, weighting, and phylogenetic utility of mitochondrial gene sequences and a compilation of conserved polymerase chain reaction primers. *Annals of the Entomological Society of America*, 87, 651-701.

Winkler, I.S., Scheffer, S.J., Mitter, C. (2009). Molecular phylogeny and systematics of leaf‐mining flies (Diptera: Agromyzidae): delimitation of *Phytomyza* Fallén sensu lato and included species groups, with new insights on morphological and host‐use evolution. *Systematic Entomology,* 34, 260-292.
